# CO_2_ Separation by Imide/Imine Organic Cages

**DOI:** 10.1002/chem.202201631

**Published:** 2022-07-25

**Authors:** Sonia La Cognata, Riccardo Mobili, Chiara Milanese, Massimo Boiocchi, Mattia Gaboardi, Donatella Armentano, Johannes C. Jansen, Marcello Monteleone, Ariana R. Antonangelo, Mariolino Carta, Valeria Amendola

**Affiliations:** ^1^ Department of Chemistry University of Pavia Viale Tarquato Taramelli 12 Pavia 27100 Italy; ^2^ Centro Grandi Strumenti University of Pavia Via Bassi 21 Pavia 27100 Italy; ^3^ Elettra sincrotrone Trieste S.C.p.a. Area science park Basovizza (TS) 34149 Italy; ^4^ Department of Chemistry & Chemical Technologies University of Calabria Via P. Bucci, 13/C 87036 Rende (CS) Italy; ^5^ Institute on Membrane Technology National Research Council of Italy (CNR-ITM) Via P. Bucci 17/C Rende (CS) 87036 Italy; ^6^ Department of Chemistry College of Science Swansea University Singleton Park Swansea Wales, SA2 8PP UK

**Keywords:** carbon capture, gas separation, mixed-matrix membranes, organic cages, porous materials

## Abstract

Two novel imide/imine‐based organic cages have been prepared and studied as materials for the selective separation of CO_2_ from N_2_ and CH_4_ under vacuum swing adsorption conditions. Gas adsorption on the new compounds showed selectivity for CO_2_ over N_2_ and CH_4_. The cages were also tested as fillers in mixed‐matrix membranes for gas separation. Dense and robust membranes were obtained by loading the cages in either Matrimid® or PEEK‐WC polymers. Improved gas‐transport properties and selectivity for CO_2_ were achieved compared to the neat polymer membranes.

## Graphical abstract

**Select and separate**: Two novel dicarboxyimide/imino‐based organic cages are proposed as materials for the selective separation of CO_2_ from N_2_ and CH_4_ under vacuum swing adsorption conditions. Robust mixed‐matrix membranes for gas separation were also obtained by loading the cages in either Matrimid® or PEEK‐WC as polymers. An improvement in the gas‐transport properties is achieved compared to that of neat polymer membranes.

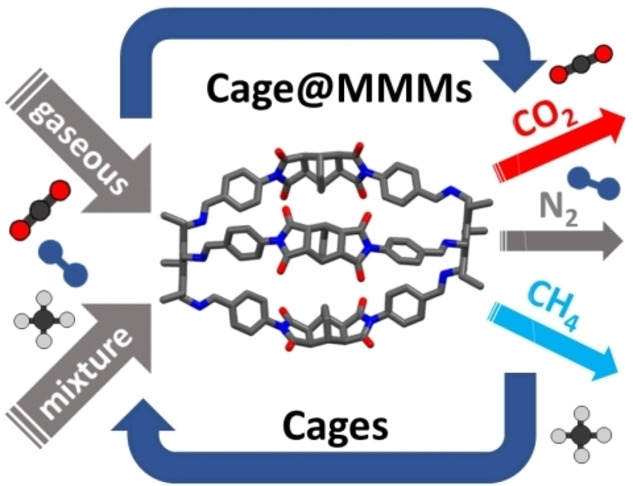

## Introduction

The growing concern about global warming is stirring worldwide interest in materials and technologies for pre‐ and post‐combustion CO_2_ capture and for clean energy production.[^1^, ^2^] Among the technologies currently applied, membrane‐based separation is more cost‐effective and energy‐efficient, but suffers from a well‐known trade‐off between permeability and selectivity.[^3^, ^4^] This point has recently been addressed by incorporating intrinsically porous materials (IPMs), selective for gases of interest, into polymeric membranes. The resulting mixed‐matrix membranes (MMMs) can boost the performance of neat membranes by combining the scalability and processability of polymers with the selectivity of fillers.^[5]^ Among the investigated IPMs, molecular materials^[6]^ – such as organic cages[^7^, ^8^] and macrocyclic compounds^[9]^ – have the advantage of being more soluble in organic solvents (compared to, e. g., metal‐organic and covalent organic frameworks)[^10^, ^11^, ^12^] and therefore more easily processable in the preparation of MMMs.^[13]^ One of the first successful attempts to obtain MMMs, using porous organic cages (POCs)^[7]^ as fillers, was achieved by crystallizing the cage molecules within the polymer membrane.^[14]^ The resulting MMMs showed enhanced permeability and resistance to physical aging. Significant enhancements of both permeability and selectivity were achieved by embedding a mixture of amorphous POCs into a composite membrane.^[15]^ In this case, a fourfold improvement in permeability was reached for CO_2_, N_2_, and CH_4_.

Over the last decades, organic cages such as azacryptands have been extensively studied in molecular recognition as selective hosts for, for example, pollutants and drugs in aqueous solution and complex matrices.[^16^, ^17^, ^18^] Following the seminal work by Cooper et al. in 2009, cages have also gained interest as porous materials for gas capture and separation. In fact, beside their intrinsic cavity, POCs can display extrinsic and interconnected pores deriving from their packing in the solid state. For gas uptake and separation applications,[^19^, ^20^, ^21^, ^22^, ^23^] POCs have been studied as either crystalline or amorphous/glassy solids,[^7^, ^24^, ^25^] and more recently in the liquid state.^[26]^ Over the last few years, POCs with different geometries (e. g., cubic,^[27]^ prismatic,^[28]^ tetrahedral[^29^, ^30^]) have been developed; in most cases, the dynamic covalent chemistry approach (e. g., imine condensation, alkyne metathesis, boronate ester formation, etc.)^[31]^ was applied for their syntheses.

The most common POCs are probably the systems obtained by imine condensation between suitably designed polyaldehydes and polyamines.[^9^, ^32^, ^33^] The [4+6] imine cages reported by Mastalerz et al.,^[8]^ for instance, display porosity features that are comparable to those achieved with some covalent organic frameworks. The selectivity for CO_2_ vs. N_2_ (or CH_4_) can be efficiently enhanced in macrocyclic hosts and cages by decorating their cavity with heteroatoms (e. g., O, N), polar groups (e. g., phenol, pyrrole, carbonyl)[^34^, ^35^, ^36^] or π‐acceptor moieties,^[37]^ that can establish hydrogen bonding or dipole‐quadrupole interactions with CO_2_. A high affinity for CO_2_ was obtained, for example, by means of urea‐based cucurbituril macrocycles.[^6^, ^38^] X‐ray diffraction studies on the hydrogen‐bonded framework generated by curcubit[6]uril molecules (CB[6]), revealed that the adsorbed CO_2_ molecules interact with the carbonyls of the CB[6] units, and establish H‐bonds with the C−H groups of CB[6] walls, exhibiting a CO_2_ heat of adsorption of 33 kJ mol^−1^.

Among porous cages, imide‐based systems recently showed interesting gas adsorption features. In particular, the soft porous crystals of the polymorphic cage obtained by condensation of a flexible polyamine with a rigid dianhydride showed a peculiar CO_2_‐induced breathing behavior.^[39]^ The interaction of CO_2_ with the cage cavity promoted the reversible switching of the cage material from a “closed” nonporous phase to a gate‐open structure. On the other hand, the imide/imine cages reported by Coskun et al.^[40]^ showed shape‐persistence in the solid state and a high affinity for CO_2_. The cage containing pyromellitic diimide units, in particular, featured a good selectivity for CO_2_ versus N_2_ (i. e., 45.5 at 273 K), as demonstrated by applying the ideal adsorbed solution theory (IAST)^[41]^ to gas‐adsorption studies results.

**Scheme 1:**
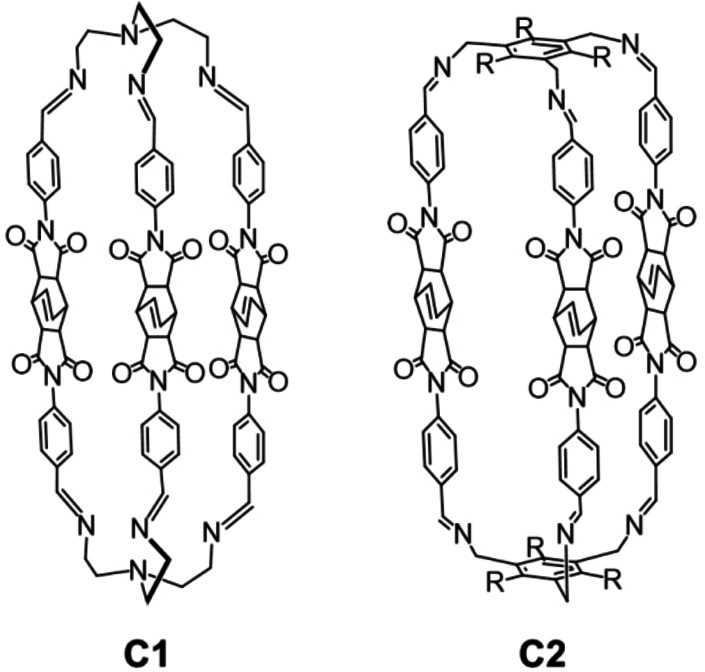
Sketches of **C1** and **C2** (R=ethyl).

In this work, we report two novel imide/imine organic cages (see **C1** and **C2** in Scheme 1), prepared by [2+3] imine condensation of two different polyamines with a novel dialdehyde compound, containing the rigid bicyclo[2.2.2]oct‐7‐ene‐2,3,5,6‐tetracarboxydiimide core. We chose to investigate this type of cages because the large number of heteroatoms and polar groups on their framework could increase the selectivity for CO_2_ with respect to N_2_ and CH_4_. We employed the bicyclic bridge‐based dialdehyde for the synthesis because its bent structure could have a positive effect on the formation of a cage, disfavoring the possible formation of oligomers/polymers (this was confirmed by our results). Gas‐adsorption studies on the cage materials showed a good selectivity for CO_2_ over N_2_ (for carbon capture and storage) and CH_4_ (for biogas upgrading) at 1–5 bar, which are the typical pressures employed during vacuum swing adsorption (VSA). The cages were also tested as fillers in MMMs, using either Matrimid® or PEEK‐WC as the polymer matrix. Preliminary studies with CO_2_, N_2_, and CH_4_ pointed out an improvement of the gas‐transport properties of the MMMs compared to the neat polymer membranes.

## Results and Discussion

### Synthesis and characterization

**C1** and **C2** were synthesized by imine condensation of either *N*,*N*‐bis(2‐aminoethyl)ethylenediamine (tren) or the 1,3,5‐tris(aminomethyl)‐2,4,6‐triethylbenzene (for **C1** and **C2**, respectively) with a bis(dicarboximide)‐based dialdehyde (see details in the Supporting Information). Both cages precipitated as pure solids from the reaction mixture in acetonitrile (MeCN) as a solvent, and the structures were confirmed by ^1^H and ^13^C NMR spectroscopies (see Figures S28‐S38). Elemental analysis allowed us to determine the formula of the two compounds: C_90_H_78_N_14_O_12_ ⋅ 4 H_2_O for **C1**; C_108_H_96_N_12_O_12_ ⋅ 2 H_2_O for **C2**.

Scanning electron microscopy (SEM) images suggest that the **C1** cage precipitated as a mixture of an amorphous material in nanometric form and larger prismatic crystals (Figure S1, while **C2** showed a crystalline prismatic well‐defined morphology (Figure S5). Higher crystallinity of **C2** compared to **C1** was confirmed by powder X‐ray diffraction (PXRD) analysis (Figures S2 and S6).

The Fourier transform infrared spectroscopy‐attenuated total reflectance (FTIR‐ATR) spectra of the two compounds show the typical C=N stretching features at 1644 and 1636 cm^−1^ respectively for **C1** and **C2** (Figures S3 and S7). Peaks assigned to the diimide groups (i. e., symmetric and asymmetric stretching of C=O) appear at 1704 and 1774 cm^−1^ for **C1**, 1709 and 1779 cm^−1^ for **C2**.

The thermal stability of **C1** and **C2** was assessed through thermogravimetric analysis (TGA). Apart from a small weight loss for the **C1** sample, very likely from humidity, **C1** and **C2** showed decomposition only starting from around 220 and 330 °C, respectively (Figures S4 and S8).

### Single‐crystal X‐ray diffraction (SCXRD) studies

Single crystals suitable for XRD study were obtained for both **C1** and **C2**.

Crystals of the compound of formula C_90_H_78_N_14_O_12_ ⋅ 2MeCN ⋅ H_2_O crystallize in the monoclinic system (**C1**‐α, *P*2_1_; *a*=19.1383(13) Å, *b*=11.6804(8) Å, *c*=19.3415(14) Å, *β*=92.2030(10)°, *V*=4320.5(5) Å^3^) and were formed by slow evaporation of a solution of **C1** in MeCN.

**Figure 1:**
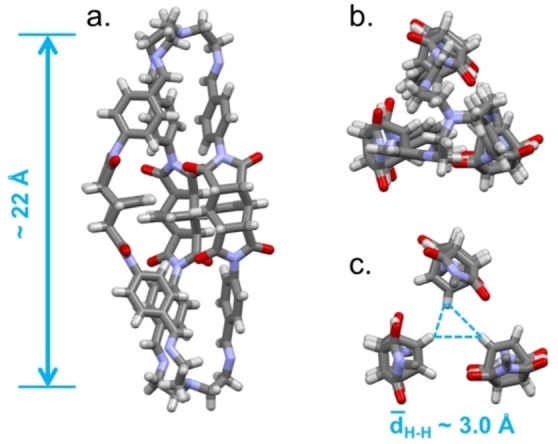
a) Front and b) top views of the crystal structure of **C1** in **C1**‐α crystals; MeCN and water molecules are omitted for clarity. c) Focus on the central portion of the cage cavity (top view), dashed cyan lines show minimum distances between HC=CH hydrogens.

As revealed by the SCXRD analysis of this crystalline **C1**‐α phase (see the Supporting Information for details), each molecular cage assumes an elongated conformation with collapsed cavity (Figure 1). The distance between the apical tertiary amines measures approximately 22 Å, and the bicyclo[2.2.2]oct‐7‐ene containing groups are oriented so as to point their HC=CH towards the cavity centre. The average shortest intramolecular separation between the HC=CH hydrogens of the three cage‐arms is about 3.0 Å. This closed conformation is stabilized by intramolecular H‐bonding interactions between carbonyl oxygens and hydrogen atoms (aryl‐H; Figure S9) of different cage arms. The asymmetric unit contains three crystallization solvent molecules (two acetonitrile and one water), as shown by the formula, that occupy extrinsic pores.

**Figure 2:**
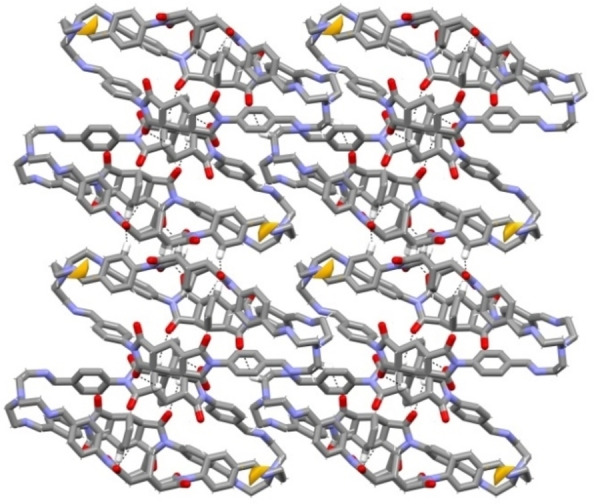
A view of the packing along the *b* crystallographic axis, for the **C1**‐α phase (MeCN and crystallization water molecules have been omitted for clarity). The free volume, as determined by the solvent‐accessible surface for a molecular probe of 1.72 Å radius, is reported in yellow.^[43]^ H‐bonding interactions are highlighted as dashed lines. H atoms are drawn as small sticks, except for the ones involved in C−H⋅⋅⋅O hydrogen bonds.

The cages are tightly packed, generating a thick network of intermolecular H‐bonds (Figure 2). These involve the carbonyl oxygens as H‐acceptors, and the C_aryl_‐H and C${}_{\mathrm{sp}{}^{3}}$ −H bonds of bicyclo[2.2.2]oct‐7‐ene‐based spacers as H donors. Geometrical features of these weak C−H⋅⋅⋅O interaction are reported in Table S2. Considering the crystal structure without guest molecules, the calculated voids accessible to a molecular probe of 1.72 Å radius (generally used for CO_2_)^[42]^ only correspond to 0.1 % of unit cell volume (Figure 2, see the Supporting Information for details).^[43]^ The calculated PXRD pattern of **C1**‐α was compared to the experimental one acquired on the microcrystalline powder of formula C_90_H_78_N_14_O_12_ ⋅ 4 H_2_O, precipitated from the reaction mixture, and used in the studies with gases (Figure S2). Although the calculated diffraction peaks of **C1**‐α can be recognized in the experimental pattern, this is dominated by the “halo” of an amorphous component. Undoubtedly, the effect of the lattice solvent plays a crucial role in supporting the ordered crystal packing, anyway we can speculate that the phase of the bulk solid corresponds to the structure reported in Figures 1 and 2.

**Figure 3:**
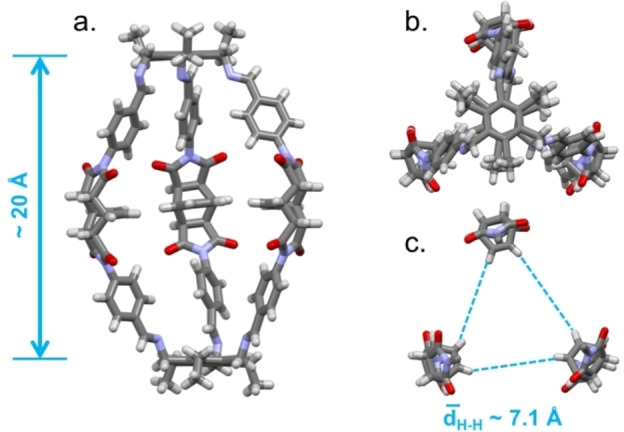
a) Front and b) top views of the crystal structure of **C2** in **C2**‐β; DMSO and water molecules are omitted for clarity; c) Focus on the central portion of the cage cavity (top view), dashed cyan lines show minimum distances between HC=CH hydrogens.

In the case of **C2**, by slow cooling of a saturated solution of the cage in dimethyl sulfoxide (DMSO), we obtained crystals corresponding to the formula C_108_H_96_N_12_O_12_ ⋅ 11 DMSO ⋅ 5 H_2_O which crystallized in the monoclinic system (**C2**‐β, P2_1_/*n*; *a*=24.354(3) Å, *b*=20.120(2) Å, *c*=31.705(3) Å, *β*=109.434(5), *V*=14650(3) Å^3^; Figure 3. As the crystals proved very fragile and unstable outside of their mother liquor, especially at room temperature, SCXRD data were collected at 190 K. Any attempt to measure them at lower temperatures (in the range 90–170 K) failed due to the collapse of the crystalline lattice. The crystal structure (see below) finally unveiled that the very large amount of lattice DMSO molecules was most likely at the origin of the intrinsic fragility. A thermal shock originates the collapse of the crystalline phase in consequence to the failure of the required fast ordered rearrangement in the supramolecular motifs involving DMSO. Analysis of the data showed that, compared to the empty and collapsed **C1** cage, the cavity of **C2** is open, large, and filled with crystallization molecules. The cage has an elongated prolate spheroid shape with a separation between the triethylaryl platforms of about 20 Å (calculated by the centroid‐centroid distance). The HC=CH bonds of the bicyclo[2.2.2]oct‐7‐ene containing spacers point towards the cage cavity, and the average separation between the HC=CH hydrogens measures 7.1 Å. In the **C2**‐β phase, adjacent cages are involved in weak C−H⋅⋅⋅O interactions having the carbonyl oxygens as H‐acceptors, and C_aryl_−H, C${}_{\mathrm{sp}{}^{2}}$ −H, and C${}_{\mathrm{sp}{}^{3}}$ −H as H‐donors; their geometrical features are reported in Table S2. However, the cage packing is less dense compared to **C1**‐α and, as previously mentioned, many additional guest solvent molecules co‐crystallized within the pores. In fact, the unit cell totally contains 16 crystallization solvent molecules (11 DMSO, 5 H_2_O), which is significant compared to the three found in the unit cell of **C1**‐α (2 MeCN, 1 H_2_O).

**Figure 4:**
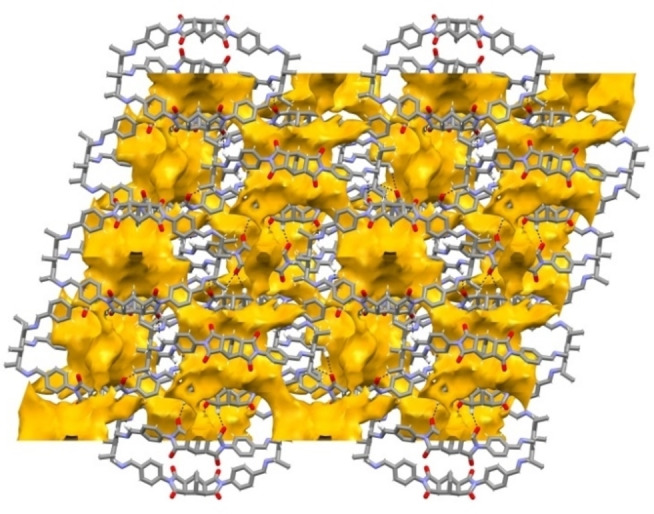
A view of the packing along the *b*‐axis for the **C2**‐β phase, obtained by omitting DMSO and water crystallization molecules. The free volume is reported in yellow, as calculated by the solvent‐accessible surface for a molecular probe of 1.72 Å radius; H‐bonding interactions are highlighted by dashed lines. H‐atoms are drawn as small sticks, except for the ones involved in C−H⋅⋅⋅O hydrogen bonds.

For the crystal structure without guest molecules, the calculated voids correspond to 9.8 % of unit cell volume (Figures 4 and S10).^[43]^ It is worthwhile mentioning that, as expected, the calculated PXRD pattern for the **C2**‐β phase is clearly different from the experimental one (Figure S6), recorded on the microcrystalline phase of formula C_108_H_96_N_12_O_12_ ⋅ 2 H_2_O (from now on called **C2**‐γ).

**Figure 5:**
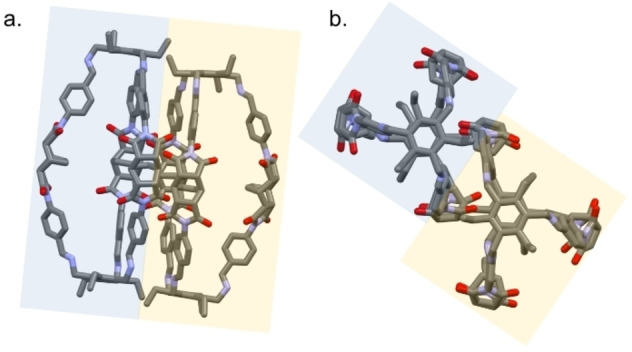
a) Front and b) top views of the two cage molecules per asymmetric unit (highlighted in blue and yellow) of the **C2**‐δ phase. Hydrogens and solvent molecules are omitted for clarity.

New crystals, suitable for SCXRD, were also obtained from a saturated solution of **C2** in MeCN. Single‐crystal data were collected at 300 K on the XRD2 beamline^[44]^ at the Elettra synchrotron light‐source (Trieste, Italy) at a wavelength of 0.6199 Å. In this solvent, **C2** crystallizes forming a triclinic phase (hereafter **C2**‐δ) containing two molecules as asymmetric unit (*Z*=6; s.g. *P* $\bar{1}$ ; *a*=14.423(3) Å, *b*=25.413(5) Å, *c*=32.957(7) Å, *α*=85.77(3)°, *β*=78.52(3)°, *γ*=78.30(3)°; *V*=11585(4) Å^3^). At 300 K, the solvent trapped in the **C2**‐δ cavities is in a disordered form. A solvent mask was calculated, and 768 electrons were found in a volume of 3648 Å^3^ in two voids per unit cell. This is consistent with the presence of about 5.8 MeCN molecules per asymmetric unit, which account for 770 electrons per unit cell, or C_108_H_96_N_12_O_12_ ⋅ 3 MeCN. The average separation between the HC=CH hydrogens is calculated to be 6.1 Å for the single molecule, with a standard deviation of 1.66 Å. However, **C2** molecules in phase **C2**‐δ form partly entangled dimers (the single cages are highlighted in blue and yellow, in Figure 5) and the effective inner radius will be smaller than that expected for a single molecule. Comparing **C2**‐β and **C2**‐δ crystal structures, it is worth noting that the latter crystallizes with a cage molecule (yellow in Figure 5) exhibiting one of the external CH_3_ groups oriented facing toward the inner void, while the structure of the second molecule (blue in Figure 5) is like that of **C2**‐β. In addition, the cage molecules are more densely packed in **C2**‐δ than in the **C2**‐β phase, and the calculated voids potentially accessible to CO_2_ are reduced to 1.9 % of unit cell volume (Figure S11). As found for **C2**‐β, the calculated PXRD pattern is different from the experimental one recorded on **C2**‐γ (Figure S6).

### Gas‐adsorption studies on C1 and C2

Gas‐adsorption studies were performed on **C1** and **C2** using CO_2_, N_2_, and CH_4_ as probe gases. As already anticipated, the **C1** solid, as precipitated from the reaction mixture, was principally amorphous. On the other hand, **C2** was collected as a microcrystalline powder, **C2**‐γ.

Before performing gas sorption studies, **C1** and **C2** samples were homogenized in a ball‐mill, and then activated by heating overnight at 350 K under vacuum to remove traces of humidity.

Brunauer‐Emmett‐Teller surface areas (SA_BET_) were initially determined using N_2_ as the probe gas. The isotherm curves showed little N_2_ adsorption at 77 K (Figures S17 and S18). The BET surface areas were calculated at a relative pressure *p/p_0_* <0.1 showing i) an apparent surface area of ∼44 m^2^ g^−1^ and a pore volume of 3.4×10^−2^ cm^3 g−1^ for **C1**; ii) an apparent surface area of ∼35 m^2^ g^−1^ and a pore volume of 8.2×10^−2^ cm^3 g−1^ for **C2**. The interpretation of the isotherms and the data suggest that both materials are either nonporous or that the pores are buried and not readily accessible, especially at 77 K, where the adsorption kinetic is very slow. The pronounced hysteresis may support the second option (Figures S12 and S13).[^45^, ^46^] The N_2_ adsorption was also measured at room temperature (298 K). This is important when used to calculate the selectivity over CO_2_, to simulate post‐combustion conditions that are normally reported at room temperature. As expected, the adsorption at this temperature is much lower than that at 77 K, with relatively low or almost no adsorption up to 1 bar (Figure S14).

To assess the affinity for CO_2_ and the separation selectivity over N_2_ and CH_4_, the CO_2_ uptake was measured at both 273 and 298 K. The lower temperature is also crucial to assess the pore size distribution (PSD)[^46^, ^47^] by both nonlocal density functional theory (NLDFT) and Horvath‐Kawazoe (H−K) methods. NLDFT is considered one of the most reliable methods to evaluate PSD, and certainly the one that gives more information about the contribute of each different pore size. PSD is preferably assessed by CO_2_ adsorption at 273 K over N_2_ at 77 K, as the latter cannot penetrate pores smaller than 5 Å whereas CO_2_ allows the evaluation of pores ∼3.5 Å, which is considered as the ultra‐microporous region.[^46^, ^48^, ^49^]

At 273 K, **C1** and **C2** samples exhibited a CO_2_ uptake of ∼17.9 cm^3^ g^−1^ (0.799 mmol g^−1^) and ∼26 cm^3^ g^−1^ (1.161 mmol g^−1^), respectively. Despite these values not being very high, the uptake is much larger than what we expected from the low N_2_ adsorption. The CO_2_ isotherms (Figures S15 and S16) show a less pronounced hysteresis than the N_2_ curves. This suggests that the materials are somewhat porous after all, but it also confirms that the pores may be poorly accessible to be measured with N_2_. It is also possible that the higher temperature (273 K for CO_2_ vs. 77 K for N_2_) produced some swelling of the flexible parts of the cages, which permits a higher and a faster kinetic adsorption of CO_2_ compared to N_2_.[^50^, ^51^] The potential deformation of the cages may also account for the relatively good CO_2_ adsorption for **C1**, which seemed unlikely by simply looking at the volume calculated by crystal structure analysis.

The calculation of the BET surface area from the CO_2_ curve is more unusual than from N_2_, but feasible and reliable.^[50]^ It showed SA_BET_ of 224 m^2^ g^−1^ for **C1** and 325 m^2^ g^−1^ for **C2** (at 273 K). These results are in line with those obtained in similar conditions by Sessler et al. for cryptand‐like cages, containing multi‐pyrrolic units as structural components (Table S3).^[34]^

The measurements of CO_2_ at 298 K showed the expected reduction in the uptake (∼13.6 cm^3^ g^−1^, 0.607 mmol g^−1^ for **C1**; ∼15.5 cm^3^ g^−1^, 0.692 mmol g^−1^ for **C2**) but the decrease is not as steep as we expected, which may suggest some contribution from chemisorption. This could be easily due to the presence of heteroatoms, polar and H‐bonding groups in the structures, which are known to enhance the affinity for CO_2_.^[39]^

Heats of adsorption (*Q*_st_) were calculated from the CO_2_ isotherms measured at 273 and 298 K. The curves were fitted with the Langmuir‐Freundlich equation and the *Q*_st_ calculated using the Clausius‐Clapeyron equation^[52]^ at zero coverage. The data showed approximate values of 35 KJ mol^−1^ for **C1** and 39 KJ mol^−1^ for **C2**, which suggests that the main adsorption mechanism is driven by physisorption, although we cannot exclude a little contribution from chemisorption (which is considered for values >30 KJ mol^−1^). Notably, these *Q*_st_ values are comparable to those obtained with cucurbituril macrocycles,^[6]^ and are indicative of the high affinity of the investigated materials for CO_2_.

The calculation of the pore size distribution by NLDFT from CO_2_ at 273 K, reveals that both materials show a series of pores centered at 3.5, 5.0 and 8.2 Å (see the Supporting Information for details). In the case of **C1**, the very small peak at ∼3.5 Å suggests little ultra‐microporosity. The low overall pore volume centered at that height (<0.1 cm^3^ nm^−1^ g^−1^) confirms the relatively low surface area. For **C2**, the peak at ∼3.5 Å is more pronounced, sign of a slightly higher ultra‐microporosity contribution. Despite the pore size distribution is considered as merely qualitative, the scattered distribution of peaks and the low amount of ultra‐micropores confirm that both materials are not very porous and that the accessibility for the gases may be hindered. The H−K model showed only the main peak centered at ∼6 Å. The peak is cut after that range, which is typical of the H−K measurements with CO_2_ at 1 bar of maximum pressure and exposes the limitations and the lack of information of this calculation over the more reliable NLDFT.

**Figure 6:**
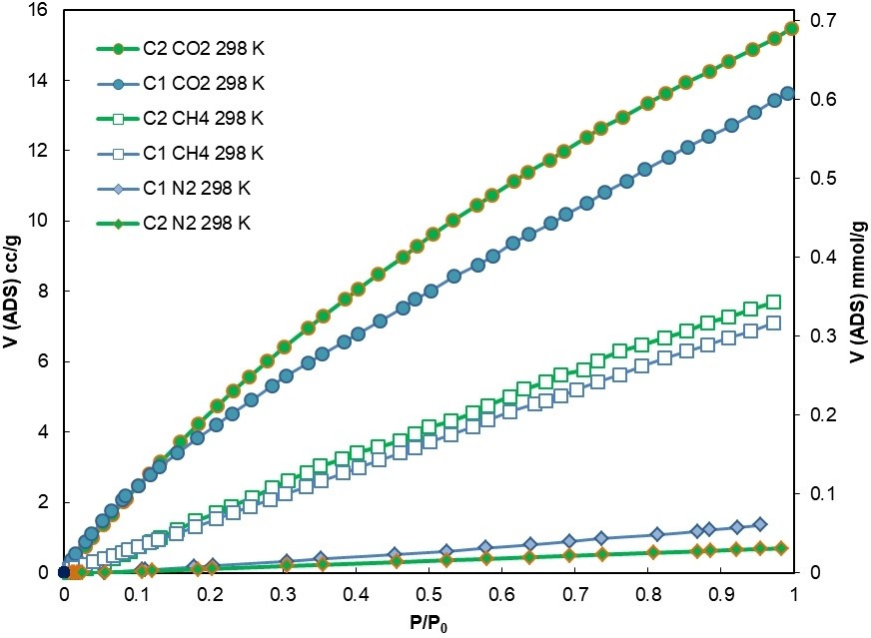
Overlay of CO_2_, CH_4_, and N_2_ adsorption isotherms, measured at 298 K. **C1** (blue) and **C2** (green). *p*_0_=1 bar, which is the maximum pressure reached by the instrument.

Comparison of the curves derived from the isothermal gas adsorption measurements at 298 K for CO_2_, N_2_ and CH_4_, provides interesting insight into the potential use of these materials for post‐combustion separation (Figure 6).^[53]^ The amount of CO_2_ adsorbed largely exceeds the amount of N_2_. Even just visually, we can expect **C2** having a better selectivity than **C1**, but the calculation for a possible post‐combustion separation cannot be done simply looking at the final uptake of the two gases at 1 bar. The first step of the calculation of the selectivity of a hypothetical CO_2_/N_2_ mixture is done by fitting the curves with the IAST method.^[41]^ In our case they were fitted from a dual‐site Langmuir‐Freundlich equation using the IAST++ software.^[54]^

The second step consists of the calculation of the ideal selectivity, assuming a 15 % CO_2_ and 85 % N_2_ gas mixture, which simulates the typical flue gas composition that is used to separate CO_2_ and N_2_ during post‐combustion carbon capture using VSA.^[55]^ The final selectivity is taken from the curve approaching 1 bar, as this is the typical pressure for post‐combustion by VSA and the maximum that our instrument can reach (Figures S23 and S24).

The selectivity of **C1** and **C2** for this separation demonstrated competitive compared to similar reported cages (Table S3) and other porous materials.[^6^, ^56^] Analysing the data, we observed that the **C1** material is slightly more selective than **C2** (41 vs. 32, Table 1). This is most likely due to its smaller pores and lower BET surface that leads to an improved molecular sieving effect. The two solids were also tested for the potential separation of CO_2_ from CH_4_, simulating biogas upgrading (i. e., the removal of CO_2_ from a mixture to improve the efficiency of CH_4_ as a fuel), which can be also performed by VSA^[57]^ at 1 bar. According to the operating conditions typically reported for this separation, the CO_2_/CH_4_ selectivity was simulated assuming a 50/50 %_v_ composition. Usually, the selectivity for this gas pair is lower than the correspondent CO_2_/N_2_ for the same materials.^[58]^ This trend is confirmed by the results herein obtained, as **C1** proved again more selective than **C2** but with much lower values compared CO_2_/N_2_ (5.3 for **C1** vs. 3.0 for **C2**).

**Table 1:** CO_2_ adsorption studies on **C1** and **C2**.

Cage	SA${}_{\mathrm{BET},\;\mathrm{CO}{}_{2}}$ ^[a]^ [m^2^ g^−1^]	*V* ${}_{\mathrm{ADS},\mathrm{CO}{}_{2}}$ ^[b]^ [cm^3^ g^−1^]	*V* ${}_{\mathrm{ADS},\mathrm{CO}{}_{2}}$ ^[c]^ [cm^3^ g^−1^]	*Q*_ST_ [kJ mol^−1^]	CO_2_/N_2_^[d]^ (IAST)
**C1**	224	17.9	13.6	35	41
**C2**	325	26.0	15.5	39	32

[a] BET surface areas (SA_BET_) calculated for CO_2_ at 273 K over the *p*/*p*_0_ range 0.01–0.1; [b] and [c] CO_2_ uptake at 273 and 298 K, respectively (1 bar). [d] selectivity of CO_2_ over N_2_ adsorption (IAST, 298 K for a CO_2_/N_2_ 15 : 85 composition).

### C1 and C2 as fillers in MMMs

**Scheme 2:**
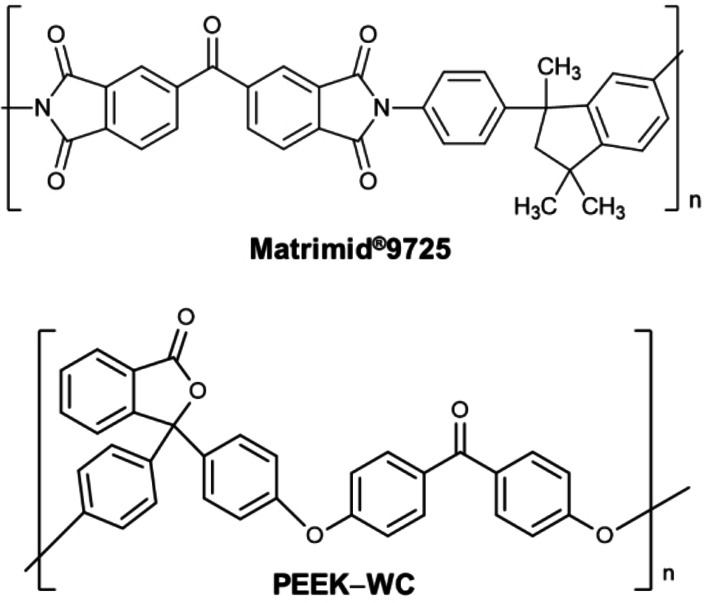
Chemical structures of Matrimid®9725 and PEEK‐WC.

The selectivity results achieved with the IAST simulation encouraged us to verify the possibility of employing our cages as fillers for MMMs for gas separation. With this is in mind, we tested our novel materials with two different polymer matrices, the commercial polyimide Matrimid® 9725 and poly(ether‐ether ketone) with cardo group, PEEK‐WC, that are both commonly used polymers for gas separation membranes (Scheme 2).[^59^, ^60^]

For the preparation of MMMs, we followed the experimental procedure recently employed by our group in the development of MMMs containing azacryptands as fillers.^[61]^ In particular, the cages were firstly homogeneously dispersed in chloroform, then the required amount of polymeric solution (2 wt.% Matrimid® 9725 or 3 wt.% PEEK‐WC in chloroform) was added. **C1** and **C2**, were loaded in the polymer matrix at 20 wt. % based on the total mass of the membranes.

The resulting mixture was sonicated, and then poured into a Teflon petri dish. Dense membranes were obtained by slow evaporation of the solvent at 25 °C for 24 h. The resulting MMMs were removed from the Petri dish and their top surface was coated with PDMS Elastosil M 4601 (prepolymer+crosslinker) to cover possible pinhole defects. Further details on MMMs preparation are reported in the Supporting Information, while details of the coating procedure were reported elsewhere.^[61]^

Pure gas permeation tests were performed at 298 K, and at a feed pressure of 1 bar in a fixed volume/pressure increase setup using the time‐lag method for the determination of the permeability (*P*), diffusion (*D*), and solubility coefficients (*S*). Details of the instrument and the measurement procedure were reported elsewhere.^[62]^

**Figure 7:**
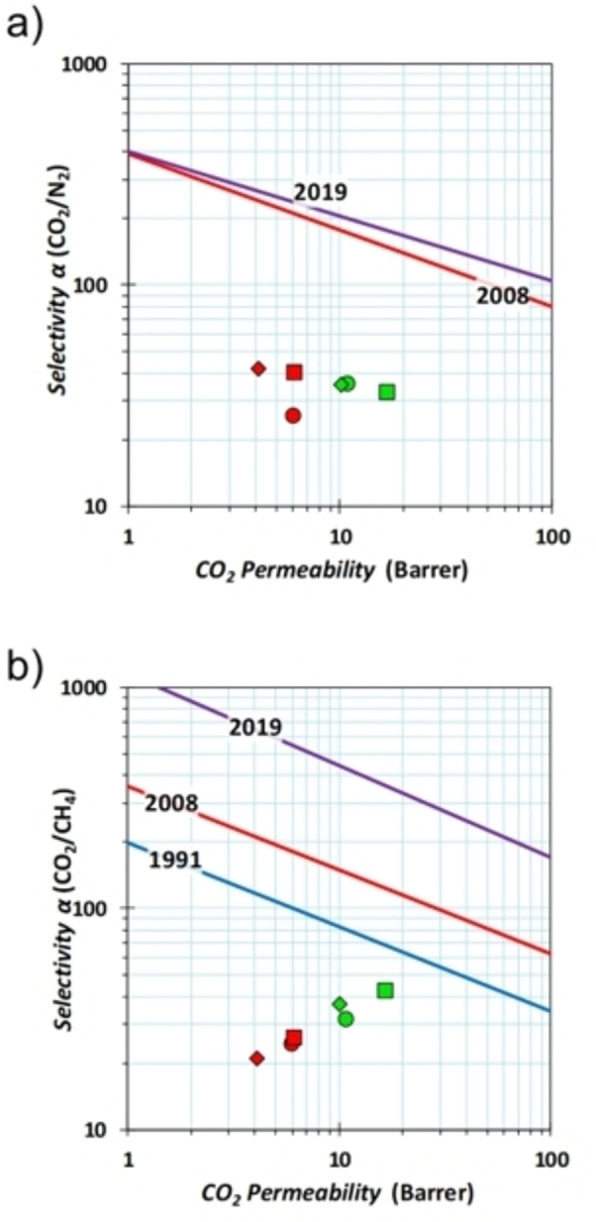
Robeson plots for the a) CO_2_/N_2_, b) CO_2_/CH_4_, gas pairs. Blue and red lines correspond to 1991 and 2008 upper bounds, respectively.[^63^, ^64^] The purple line corresponds to the 2019 upper bound.^[66]^ The data are reported as: •: Matrimid® 9725, ▪: Matrimid® 9725/**C2**, 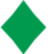: Matrimid® 9725/**C1**, •: PEEK‐WC, ▪: PEEK‐WC/**C2**, and 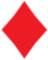: PEEK‐WC/**C1**.

The Robeson plots (Figure 7) show the permeability and selectivity data for the two most interesting gas pairs, CO_2_/CH_4_ and CO_2_/N_2_. These plots, in which, for example, the selectivity for CO_2_ versus N_2_ is plotted against the CO_2_ permeability (Figure 7a), are generally used to compare the performance of different materials with respect to the state of the art.[^63^, ^64^] In particular, the Robeson plots in Figure 7 compare the performance of our MMMs (i. e., green diamond, Matrimid® 9725/**C1**; green square, Matrimid® 9725/**C2**; red diamond, PEEK‐WC/**C1**; red square, PEEK‐WC/**C2**) and of the neat polymers (green and red circles for Matrimid® 9725 and PEEK‐WC, respectively) with the best performance achieved for a given separation, as defined by the upper bounds (see the diagonal lines in Figure 7).

Notably, **C1** and **C2** have opposite effects on the CO_2_ permeability (Table 2). In particular, the addition of **C1** as filler results in a marginal decrease of the CO_2_ permeability in Matrimid (green diamond vs. green circle in Figure 7), and an approximately 1/3 decrease in PEEK‐WC (red diamond vs. red circle). On the other hand, **C2** increases the CO_2_ permeability by few percent in PEEK‐WC (Table 2; red square vs. red circle in Figure 7) and about 50 % in Matrimid® 9725 (green square vs. green circle). On the basis of the Maxwell equation (see the Supporting Information for details), the decrease in permeability for **C1** and the increase for **C2** suggest that the fillers have a lower and higher permeability than the polymers, respectively.^[61]^ This is also in line with the lower porosity of **C1** compared to **C2**, resulting from the gas‐adsorption studies on the two materials (see, e. g., SA${}_{\mathrm{BET},\;\mathrm{CO}{}_{2}}$ values at 298 K in Table 1).

**Table 2:** Pure gas permeability and selectivity for neat and mixed matrix membranes.

Membrane	Permeability (barrer)			Selectivity *α*(*P*_x_/*P*_y_)	
	N_2_	CH_4_	CO_2_	CO_2_/N_2_	CO_2_/CH_4_
PEEK‐WC^[65]^	0.24	0.25	6.04	25.4	23.9
20 % **C1**	0.10	0.20	4.10	42.1	20.8
20 % **C2**	0.15	0.24	6.15	39.9	25.7
Matrimid®9725	0.31	0.35	10.8	35.5	31.1
20 % **C1**	0.28	0.28	10.1	35.4	36.6
20 % **C2**	0.51	0.40	16.7	32.7	41.7

As for selectivity, both **C1** and **C2** significantly increase the CO_2_/N_2_ selectivity in PEEK‐WC (Table 2 and Figure 7a). Improved CO_2_/CH_4_ selectivity is also found for the PEEK‐WC/**C2** membrane (Figure 7b). Noticeably, both cages increase the CO_2_/CH_4_ selectivity in Matrimid® 9725 but did not affect the CO_2_/N_2_ selectivity (**C1**) or slightly decrease it (**C2**).

These changes are associated with a general increase of the effective diffusion coefficient by **C2** in both polymers (Table 3), especially for the gas with small molecular diameter. Instead, **C1** increases the diffusion coefficient in PEEK‐WC, but reduces it in Matrimid® 9725.

**Table 3:** Diffusion coefficients and solubility for neat and mixed matrix membranes.

Membrane	Diffusivity [10^−12^ m^2^ s^−1^]			Solubility [cm^3^_STP_ cm^−3^ bar^−1^]		
	N_2_	CH_4_	CO_2_	N_2_	CH_4_	CO_2_
PEEK‐WC^[65]^	0.45	0.14	0.58	0.39	1.33	7.77
20 % **C1**	0.68	0.15	0.59	0.11	0.95	5.19
20 % **C2**	0.91	0.14	0.74	0.13	1.24	6.22
Matrimid®9725	1.12	0.19	0.84	0.20	1.32	9.59
20 % **C1**	0.69	0.14	0.64	0.31	1.50	11.8
20 % **C2**	1.22	0.22	1.21	0.31	1.38	10.4

**Figure 8:**
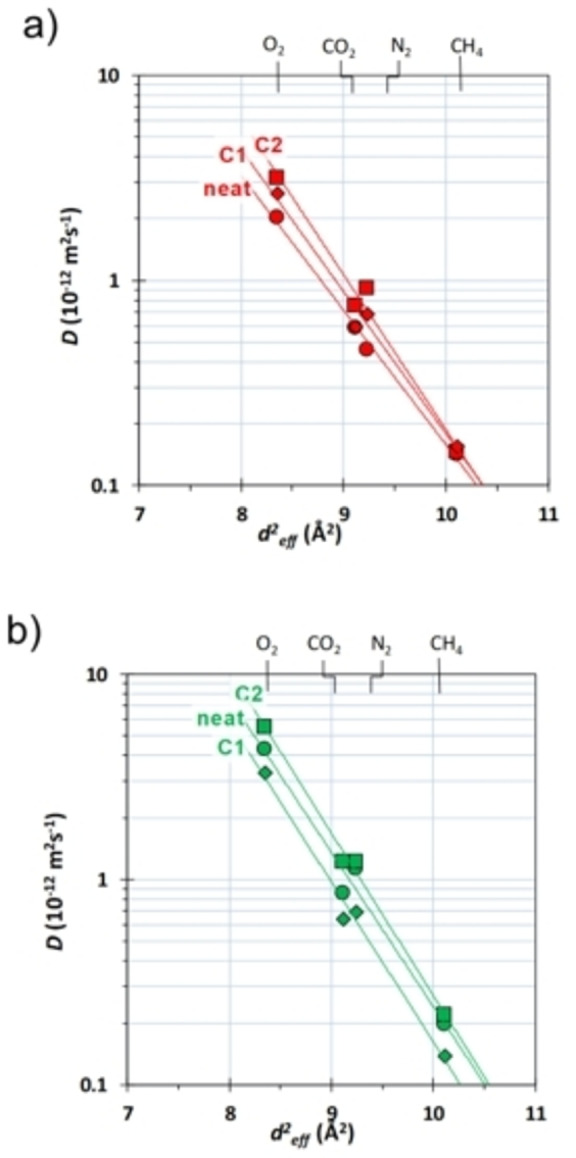
Correlation of the effective diffusion coefficient as a function of the molecular diameter^[67]^ of four light gases in a) PEEK‐WC and b) Matrimid® 9725. The various symbols are referred to: •: Matrimid® 9725, ▪: Matrimid® 9725/C2, : Matrimid® 9725/C1, •: PEEK‐WC, ▪: PEEK‐WC/C2 and : PEEK‐WC/C1.

In PEEK‐WC, these changes are also accompanied by an increase in size‐selectivity, as the slope of the correlation of log(*D*) versus the squared gas diameter (d^2^_eff_) increases with both cages (see the red lines in Figure 8a). On the other hand, the cages have negligible effect on the size‐selectivity in Matrimid® 9725 (Figure 8b). The differences between the two cages can be ascribed to the higher porosity of **C2**.

## Conclusion

Two novel imide/imine‐based organic cages are reported in this work. The systems were investigated as materials for gas‐separation processes both as pure solids and loaded in polymeric membranes.

SCXRD analyses showed that both cages crystallize to form a thick network of intermolecular H‐bonds, which principally involve the bicyclo[2.2.2]oct‐7‐ene‐based spacers (the carbonyl oxygens and the C_aryl_−H/C${}_{\mathrm{sp}{}^{3}}$ −H bonds, in particular) of different cage molecules. The thick packing significantly reduces the free volume potentially accessible to gases in the crystal. In fact, when a molecular probe of 1.72 Å radius was considered, the calculated voids represented only 0.1 and 9.8 % of unit cell volume for **C1** and **C2**, in **C1**‐α and **C2**‐β phases, respectively.

The crystalline materials could not be produced in a large enough quantity to allow us to perform studies with gases. These investigations were therefore conducted on the solids precipitated from the reaction mixtures, and then ball‐milled to achieve materials that could be homogenously dispersed into polymer matrices for the preparation of MMMs. PXRD analyses showed that the **C1** material was mostly amorphous, whereas **C2** was obtained as a microcrystalline phase, although different from those crystallized from DMSO or MeCN, and characterized by SCXRD.

Adsorption studies with N_2_ at 77 K suggest that the accessibility to pores is rather hindered in both **C1** and **C2**. This result is not surprising, considering the tight packing and the thick network of H‐bonds revealed by crystal structures.

Gas‐adsorption studies were also performed with CO_2_, N_2_ and CH_4_ at 298 K. Notably, the BET surface areas calculated in these conditions from CO_2_ resulted much higher than the values obtained with N_2_ at 77 K. We could conclude that i) the **C1** and **C2** materials are more porous than expected from studies with N_2_ at cryogenic temperature, ii) the cages framework is flexible enough to swell with increasing temperature.

**C1** and **C2** were also successfully tested for potential CO_2_/N_2_ and CO_2_/CH_4_ separations in VSA processes. The good selectivity for CO_2_ confirms that imide/imine groups strengthen the interaction with the quadrupolar CO_2_ gas with respect to N_2_ and CH_4_ species.

**C1** and **C2** also proved to be suitable as fillers for mixed‐matrix membranes. Dense and robust MMMs were obtained from both cages with both PEEK‐WC and Matrimid® 9725 polymers. Pure gas permeation tests with CO_2_, N_2_, and CH_4_ gases showed an improvement in gas‐transport properties compared to the pure polymer membranes. An increase in CO_2_/CH_4_ selectivity was found with **C1** and **C2** in Matrimid® 9725, whereas an increase in CO_2_/N_2_ selectivity was achieved with both cages in PEEK‐WC.

## Experimental Section

Deposition Numbers 2172063 (for **C1**‐α), 2172064 (for **C2**‐β), and 2172328 (for **C2**‐d) contain the supplementary crystallographic data for this paper. These data are provided free of charge by the joint Cambridge Crystallographic Data Centre and Fachinformationszentrum Karlsruhe Access Structures service.

All the experimental details are reported in the Supporting Information.

## Conflict of interest

The authors declare no conflict of interest.

1

## Supporting information

As a service to our authors and readers, this journal provides supporting information supplied by the authors. Such materials are peer reviewed and may be re‐organized for online delivery, but are not copy‐edited or typeset. Technical support issues arising from supporting information (other than missing files) should be addressed to the authors.

Supporting Information

## Data Availability

The data that support the findings of this study are available from the corresponding author upon reasonable request.
