# Supplementary material for: CO2 Separation by Imide/Imine Organic Cages
Source: Chemistry. 2022 Jul 25;28(49):e202201631. doi: 10.1002/chem.202201631 (PMC9545214; doi:10.1002/chem.202201631)
Supplement: Supplementary file 1 — Supporting Information [file CHEM-28-0-s001.pdf]

Chemistry–A European Journal

Supporting Information

CO_2 Separation by Imide/Imine Organic Cages

Sonia La Cognata, Riccardo Mobili, Chiara Milanese, Massimo Boiocchi, Mattia Gaboardi, Donatella Armentano, Johannes C. Jansen,* Marcello Monteleone, Ariana R. Antonangelo, Mariolino Carta,* and Valeria Amendola*

Index

1. Materials and Methods *S3*

2. Synthetic procedures *S5*

3. Physicochemical characterization of C1 and C2 *S8*

4. SCXRD studies *S13*

5. Gas adsorption studies on C1 and C2 *S19*

6. Single gas permeation studies on mixed matrix membranes *S31*

7. NMR spectra of new compounds *S34*

Additional references *S40*

1. Materials and Methods

All commercially available starting materials and solvents were purchased from Sigma-Aldrich and VWR, and used without further purification. 4-aminobenzaldehyde was obtained using a modified known procedure (see below). The synthesis of 1,3,5-tris(methylamino)-2,4,6-triethylbenzene is reported elsewhere.[1] All reactions were performed under nitrogen. All the samples were homogenized by ball-milling for 30 min (first cycle of 20 min + 10 min pause + second cycle of 10 min) at a milling speed of 350 rpm using a ball-milling Planetary mill (Pulverisette 7, Premium line, Fritsch, Germany) in tungsten carbide bowls (Fritsch) with WC balls (Fritsch) in a ball to powder ratio of ca. 5:1 (ball diameter 0.5 cm, ball mass ca. 1g). ^{1}H- and ^{13}C-NMR spectra were recorded on a Bruker AVANCEIII 400 MHz (operating at 9.37 T, 400 MHz), equipped with a 5 mm BBO probe head with Z-gradient (Bruker BioSpin). Deuterated solvents used for NMR analysis ($CDCl_3$, CD_3CN) were purchased and used as received. Chemical shifts are reported in ppm with the residual solvent as internal reference, while 2D spectra were graphically referenced. The HSQC spectrum for **C1** was recorded on a Bruker AVANCEIII 400 MHz, at 353K. The data were processed with Topspin 3.6 (Bruker Biospin). The HSQC spectrum for **C2** was recorded at 298K on a Bruker Avance NEO 700MHz spectrometer, equipped with a triple resonance helium cooled cryoprobe. The data were processed with Topspin 4.1 (Bruker Biospin). Elemental analyses were conducted on a Perkin Elmer CHN 2400SERIES II ELEMENTAL ANALYZER at the Department of Chemistry of the University of Milano (Italy).

1.1 Solid-state characterization of C1 and C2

For the Fourier Transform – Infrared analysis (FT-IR), a Nicolet FT-IR iS10 spectrometer (Nicolet, Madison, WI, USA) equipped with attenuated total reflectance (ATR) sampling accessory (Smart iTR with diamond plate) was used. Thirty-two scans in the 4000–600 cm^{-1} range at 4 cm^{-1} resolution were co-added. Well-ground powder samples were used, and spectra were obtained after pressing the sample onto an ATR diamond crystal at room temperature (20 °C). Peak wavenumbers were attributed by using the “Find peaks” function of the OMNIC™ Spectra Software.

Powder X-ray diffraction (PXRD) measurements were performed at room temperature on powders of the cage samples after manual grinding in an agate mortar using a Bruker D5005 diffractometer (Bruker Corporation, Billerica, MA, USA) with Cu-K$_{\alpha}$ radiation, graphite monochromator, and a scintillation detector. The measurements were performed from 5° to 50° in 2θ with step scan mode: scan step 0.02°, counting time 10 s per step; X-ray tube working conditions: 40 kV and 40 mA.

Thermogravimetric analysis (TGA) was performed by a Q5000 apparatus (TA Instruments, New Castle, DE, USA) interfaced with a TA5000 data station under nitrogen flux (10 mL min^{-1}) in a platinum pan by heating about 3 mg of sample from room temperature up to 1000 °C (heating rate 5 K min^{-1}). TGA data were analyzed by the Universal Analysis software by TA Instruments.

A Zeiss EVO MA10 (Carl Zeiss, Oberkochen, Germany) Scanning Electron Microscope (SEM) was used for the morphological study on gold sputtered samples (Secondary Electrons detector). The measurements were performed at 20 kV with a working distance of 8.5 mm.

Experimental details of crystallographic analysis are reported in Section 4.

1.2 Gas adsorption studies on C1 and C2

The sample was provided as fine crystalline/powder. It was then weighed in a Quantachrome adsorption cell and outgassed under vacuum for 15 hours at 303 K.

Low-temperature N_2 (77 K and 298 K), CO_2 (273 K and 298 K) and CH_4 (298 K) adsorption/desorption measurements of PIM powders were made using a Quantachrome Nova-e. Samples were degassed for 800 min at 100 °C under high vacuum prior to analysis. The gases were supplied by BOC and used without any further purification (N_2 purity > 99.999, CO_2 purity > 99.995%, CH_4 purity > 99.995%). The specimen was measured twice after outgas in two different cells to minimize the error, providing the same results. The data were analysed with the software provided with the instrument. The BET was calculated at a relative pressure $P/P_0 < 0.1$. NLDFT and H-K analysis were performed to calculate the pore size distribution and volume, considering a carbon equilibrium transition kernel at 273 K based on a slit-pore model; the kernel is based on a common, one centre, Lennard-Jones model. P_0 always refers to 1 bar, which is the maximum pressure reached by the instrument, not the saturation pressure of the probe gas. To assess the potential chemisorption, heats of adsorption were calculated from the CO_2 curves measured at 237K and 298K. The data were analysed with the QuadraWin software and fitted with the Langmuir-Freundlich equation and calculated via the Clausius-Clapeyron equation.

1.3 Preparation of the mixed matrix membranes

The two cages **C1** and **C2** were loaded in the polymer matrix at 20 wt % on the basis of the total mass of the membranes. The polymers Matrimid® 9725 and PEEK-WC were dissolved at 2 wt.% and 3 wt.% respectively, in chloroform for 24h, and filtered by glass syringe filter of 3.1µm. Cages **C1** and **C2**, (15 mg each for PEEK-WC membranes and 30 mg for Matrimid® 9725 based) were first dispersed in chloroform (1 g) by sonication for 30 min at 25 °C and then the required amount of polymeric solution was added to this dispersion. The resulting mixture was further sonicated for 3

hrs. to obtain a homogenous dispersion of the cages. Finally, the dispersion was poured into a Teflon petri dish and dense membranes were obtained by slow evaporation of the solvent at 25 °C for 24 hrs.. The resulting mixed matrix membranes were removed from the Petri dish and their top surface was then coated with a diluted PDMS Elastosil M 4601 (prepolymer+cross-linker) solution in n-hexane to cover possible pinhole defects. The coated membranes were kept at room temperature to complete the crosslinking in 24 hrs. Further details of the coating procedure were reported previously.[2]

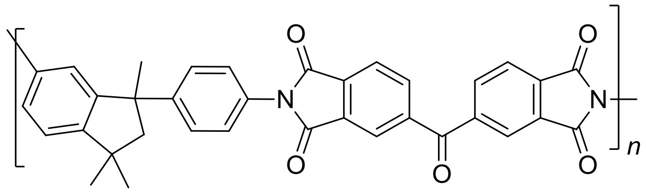

Matrimid® 9725

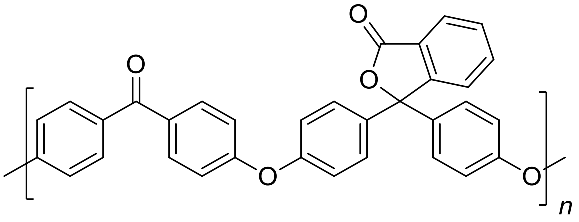

PEEK-WC

2. Synthetic procedures

2.1 Synthesis of 4-aminobenzaldehyde

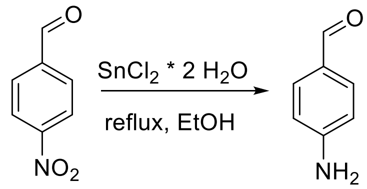

1 g of 4-nitrobenzaldehyde (6.6 mmol; 1 eqv) and 7.5 g of $SnCl_2 \cdot 2\ H_2O$ (33 mmol; 5 eqv) were added to 30 mL of EtOH and refluxed under N_2. The reaction was monitored by TLC (Silica gel, *n*-hexane:AcOEt = 1:1) until the complete disappearance of the reactant (about2 hours). The red-brown mixture was then cooled to r.t. and poured in 120 g ice. pH was adjusted to 10-12 by the addition of aqueous NaOH. The yellow aqueous mixture was then extracted with AcOEt (5 x 100 mL). The organic phases where collected, washed with brine (2 x 200 mL), dried on Na_2SO_4, filtered and the solvent was removed under vacuum to obtain 730 mg of an orange solid. The final product was used without further purification. Yield: 91.3 %. ^{1}H-NMR (400 MHz; $CDCl_3$) δ: 9.68 (s, 1H, C*H*O), 7.63-7.61 (d, 2H, CH_{ph}), 7.64-7.62 (d, 2H, CH_{ph}), 4.17 (broad, 2H, NH_2).

2.2 Synthesis of 1

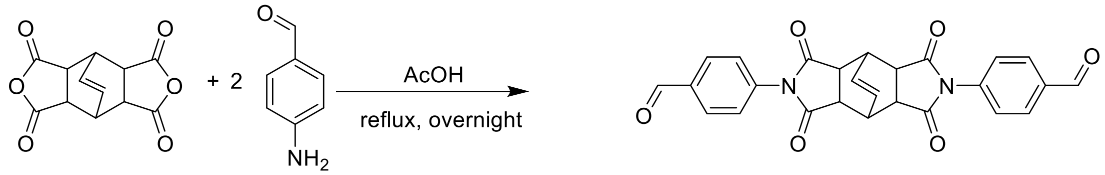

270 mg of bicyclo[2.2.2]oct-7-ene-2,3,5,6-tetracarboxylic dianhydride (1.09 mmol; 1 eqv) and 330 mg of 4-aminobenzaldehyde (2.72 mmol; 2.5 eqv) were added in a round-bottom flask to 20 mL of acetic acid (AcOH). The reaction mixture was refluxed overnight under vigorous stirring. The mixture was then cooled to r.t. and the precipitate (a light brown solid) was collected on a Buchner funnel. The solid was washed with AcOH (20 mL), H_2O (20 mL), MeOH (20 mL), Et_2O (20 mL) and dried under vacuum. The final product (340 mg, light brown powder) was used without further purification. Yield: 68.8 %. ATR-FTIR, cm^{-1}: 1699 (str. C=O), 1707 (sym. str. C=O), 1779 (asym. str. C=O). ^{1}H-NMR (400 MHz; DMSO-d6) δ: 10.04 (s, 1H, C*H*O), 8.03 (d, 2H, CH_{ph}), 7.44 (d, 2H, CH_{ph}), 6.35 (m, 1H, CH_{olef}), 3.57 (s, 1H, C*H*), 3.49 (s, 2H, C*H*). ^{13}C-NMR (100 MHz; DMSO-d6) δ: 192.88, 176.83, 137.47, 136.10, 131.63, 130.51, 127.79, 43.04, 34.52. $C_{26}H_{18}N_2O_6$ + H_2O: calc. C 66.10%, H 4.27%, N 5.93%; exp. C 66.08%, H 4.40%, N 5.89%.

2.3 Synthesis of C1

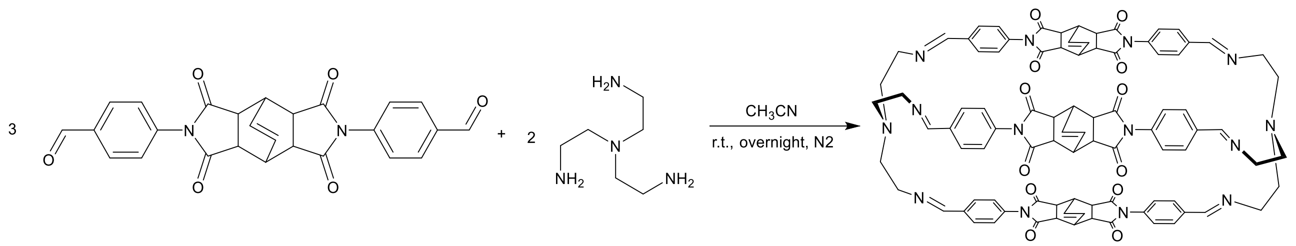

100 mg of **1** (0.22 mmol; 3 eqv) were dissolved in 100 mL of MeCN in a 3-necked round-bottom flask under N_2 and vigorous stirring. A solution of 23 μL of tris(2-aminoethyl) amine (96%; d=0.978; 0.15 mmol; 2 eqv) in 50 mL of MeCN was then added from a dropping funnel over 3 hours. The reaction mixture was then stirred overnight at r.t. under N_2. The mixture was then concentrated under vacuum and the precipitate was filtered on a Buchner funnel. The solid product was washed with MeCN (10 mL) and dried under vacuum to yield 100 mg of an off-white powder. Yield: 86.2 %. ATR-FTIR, cm^{-1}: 1644 (str. C=N), 1704 (sym. str. C=O), 1744 (asym. str. C=O). ^{1}H-NMR (400 MHz; DMSO-d6; 80 °C) δ, ppm: 7.57 (s, 1H, C*H*N), 7.12 (d, 2H, CH_{ph}), 6.94 (d, 2H, CH_{ph}), 6.29 (m, 1H, CH_{olef}), 3.63 (s br, 1H, C*H*), 3.49 (s, 4H, C*H* + C$H_{2\text{tren}}$), 2.75 (m, 2H, C$H_{2\text{tren}}$). ^{13}C-NMR (100 MHz; DMSO-d6; 80 °C) δ, ppm: 177.28, 160.15, 136.72, 134.59, 131.24, 129.18, 129.01, 59.53, 55.48,

43.19, 34.50. $C_{90}H_{78}N_{14}O_{12} \cdot 4H_2O$: calc. C 66.20%, H 5.41%, N 12.07%; exp. C 66.74%, H 5.35%, N 12.11%.

2.4 Synthesis of C2

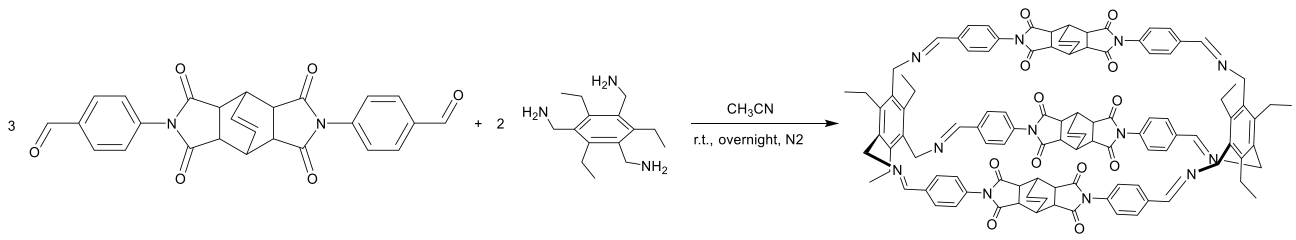

100 mg of dialdehyde **1** (0.22 mmol; 3 eqv) were dissolved in 100 mL of MeCN in a 3-necked round-bottom flask under N_2 and vigorous stirring. A solution of 36.6 mg of 1,3,5-tris(methylamino)-2,4,6-triethylbenzene (0.15 mmol; 2 eqv) in 5 ml of dichloromethane was added to 50 mL of MeCN and the resulting solution was added to the dialdehyde solution with a dropping funnel in 3 hours. The reaction was then stirred overnight at r.t. under N_2. After a night a precipitate was formed. The mixture was filtered on a Buchner funnel and the solid was washed with MeCN (10 mL) and dried under vacuum to obtain 72 mg of an off-white powder. Yield: 54.7 %. ATR-FTIR, cm^{-1}: 1636 (str. C=N), 1709 (sym. str. C=O), 1749 (asym. str. C=O). ^{1}H-NMR (400 MHz; DMSO-d6) δ:. 8.47 (s, 1H, C*H*N), 7.77 (d, 2H, CH_{ph}), 7.19 (d, 2H, CH_{ph}), 6.24 (t, 1H, CH_{olef}), 4.89 (s, 1H, CH_2N), 3.55 (s, 1H, C*H*), 3.40 (s, 2H, C*H*), 2.77 (q, 2H, CH_2), 1.20 (t, 3H, CH_3). ^{13}C-NMR (700 MHz, DMSO-d6) δ: 176.91, 160.74, 143.16, 136.42, 134.28, 132.98, 131.47, 128.81, 127.25, 58.10, 43.02, 34.20, 22.51, 13.12. $C_{108}H_{96}N_{12}O_{12} \cdot 2H_2O$: calc. C 72.46%, H 5.63%, N 9.39%; exp. C 72.43%, H 5.69%, N 9.27%.

3. Physicochemical characterization of C1 and C2

3.1 Characterization of C1

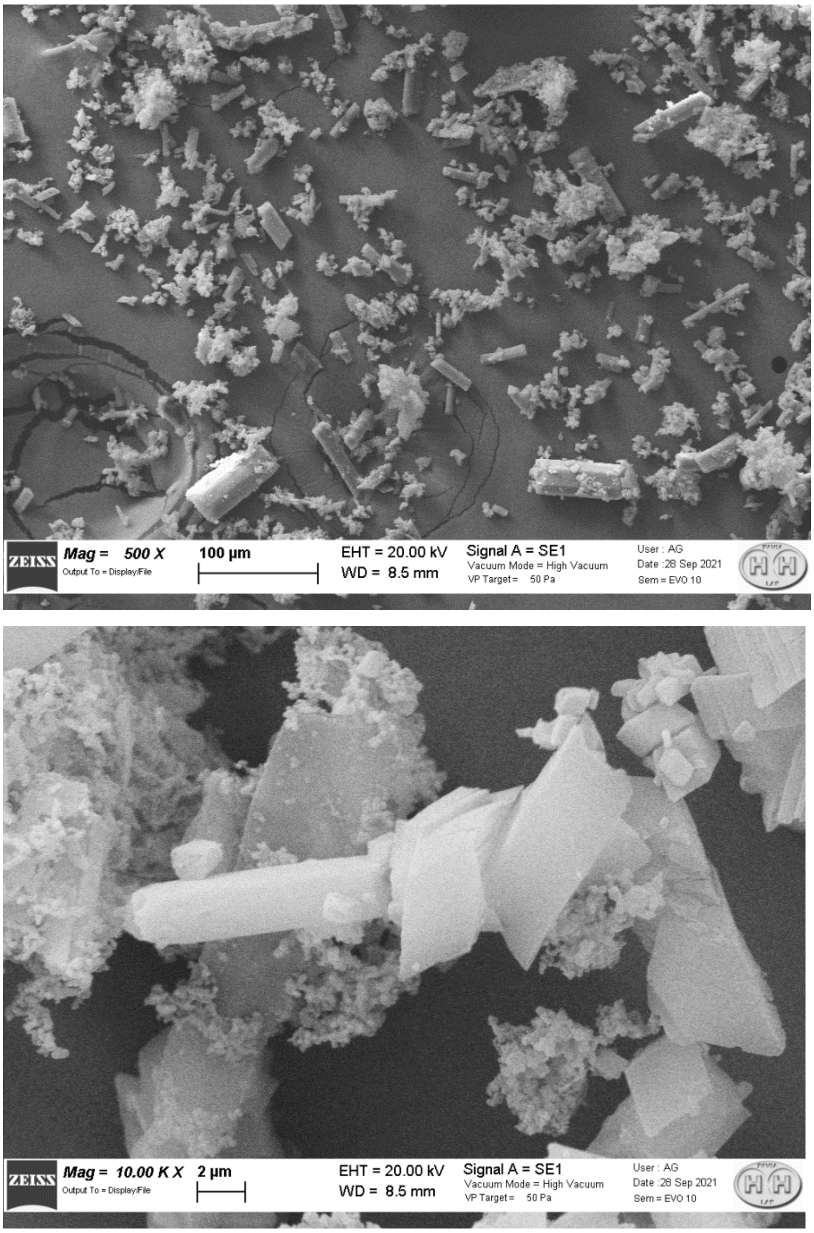

Fig. S1: SEM images of **C1** as precipitated from reaction mixture; the corresponding experimental PXRD pattern is shown in Fig. S2.

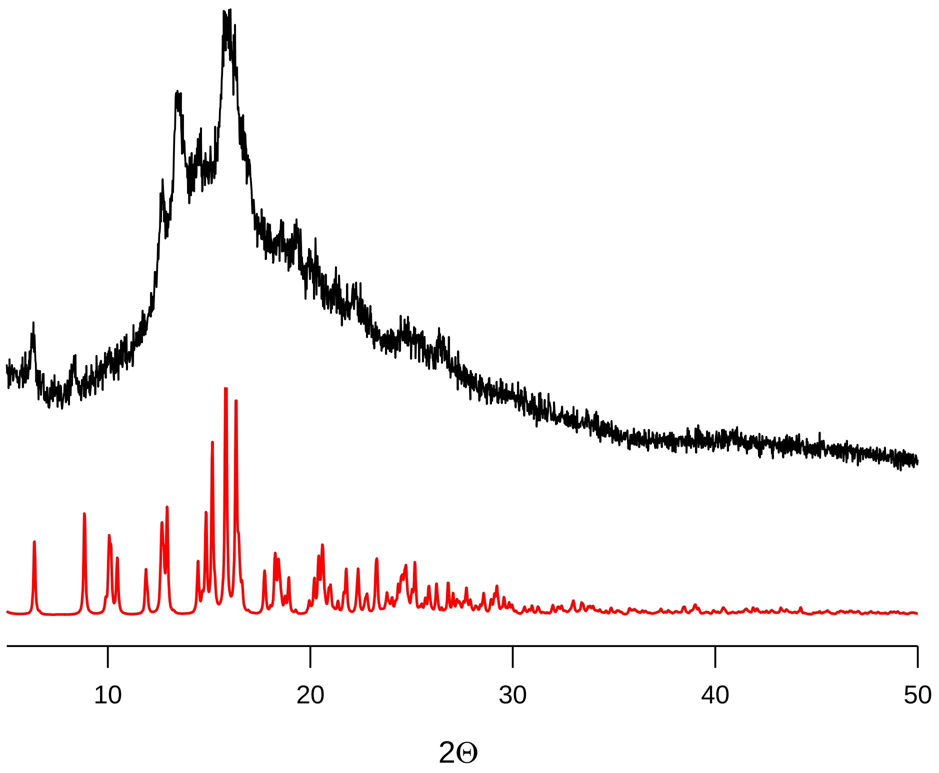

Fig. S2: Experimental PXRD pattern (black line) for **C1**, recorded on the bulk solid precipitated from the reaction mixture (MeCN solvent); simulated PXRD pattern (red line) produced using the SCXRD data collected on crystals of **C1**-α, obtained by slow evaporation of an MeCN solution of the cage. Experimental and simulated patterns were both collected at room temperature. On y axis: Intensity (a.u.); on x-axis: 2θ values.

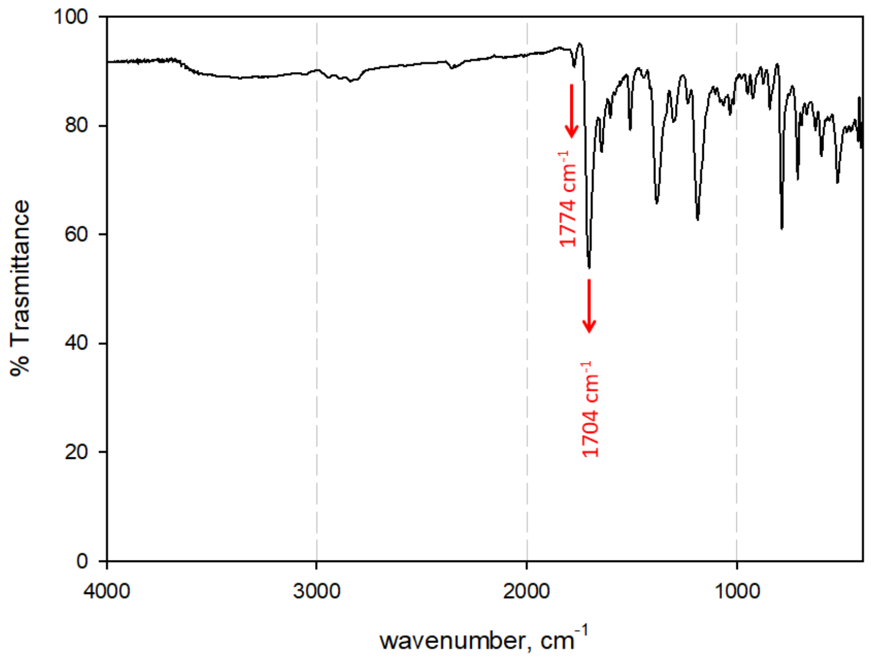

Fig. S3: ATR-FTIR spectrum of **C1**

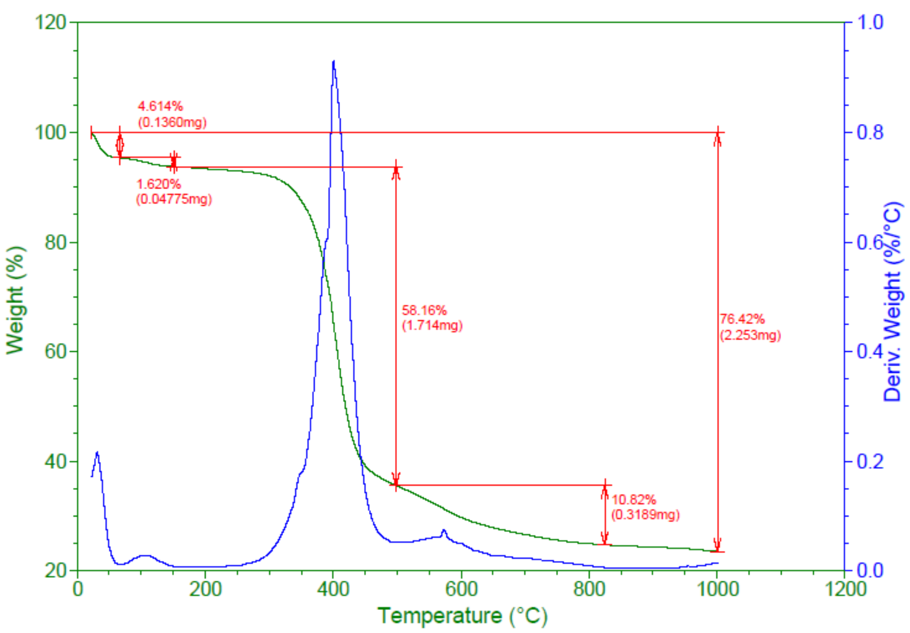

Fig. S4: TGA-DTG curves for **C1**

3.2 Characterization of C2

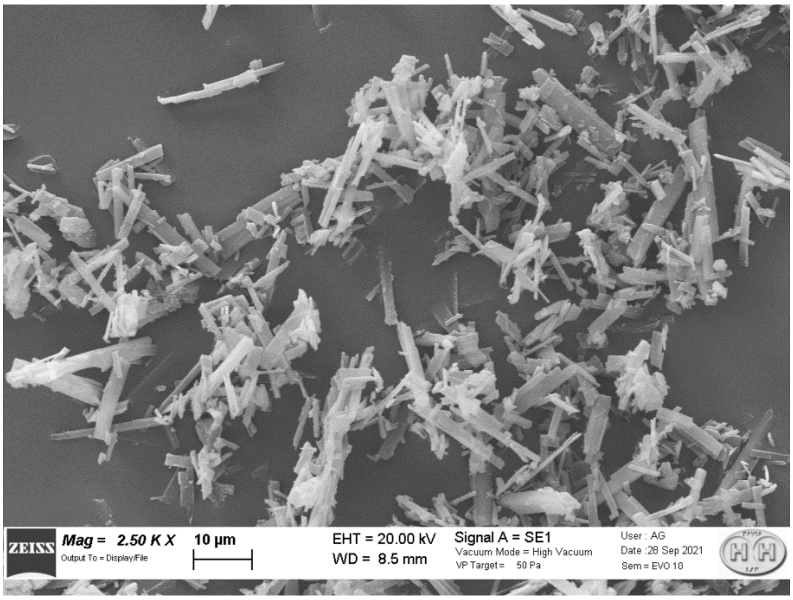

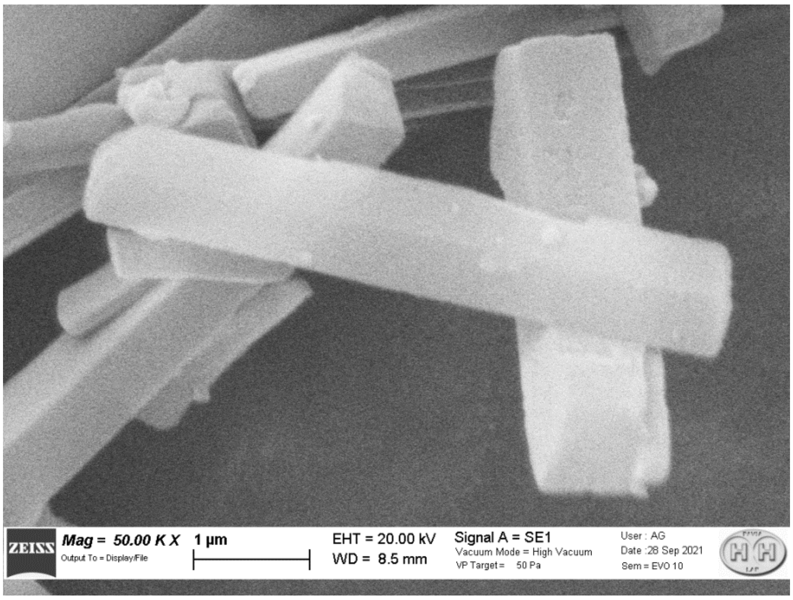

Fig. S5: SEM images of **C2** as precipitated from reaction mixture; the corresponding experimental PXRD pattern is shown in Fig. S6 (black line).

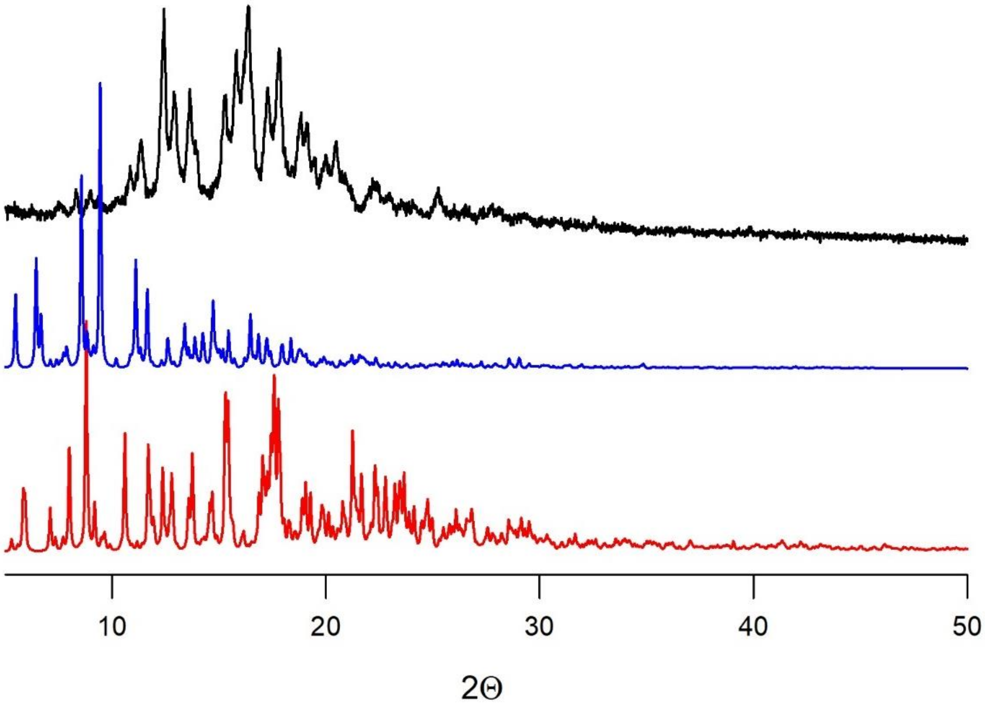

Fig. S6: Experimental PXRD pattern (black line) for **C2**, recorded on the bulk solid precipitated from the reaction mixture (MeCN solvent); simulated PXRD patterns produced using the SCXRD data collected on crystals of **C2-**β (red line) and **C2-**δ (blue line). On y axis: Intensity (a.u.); on x-axis: 2θ values.

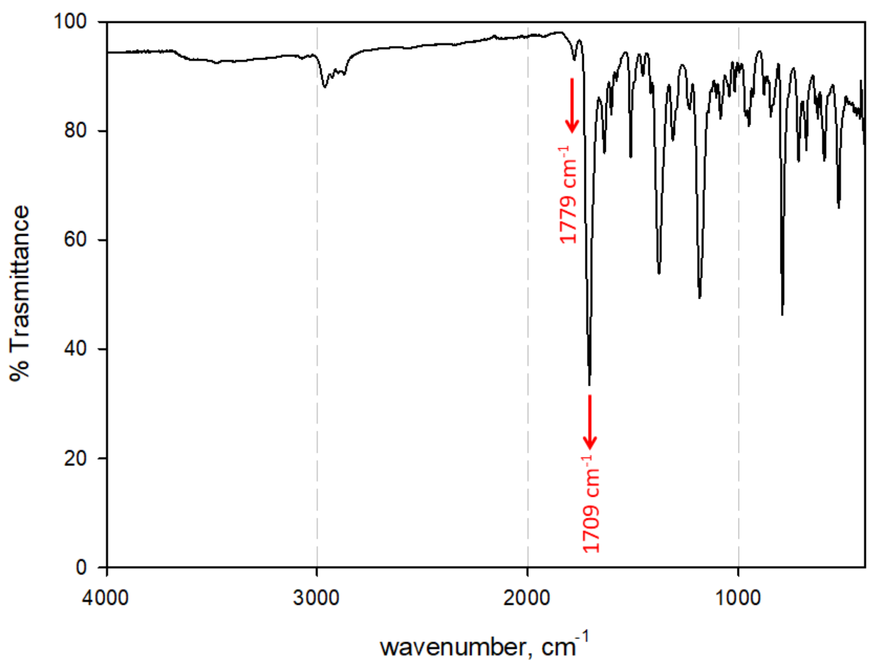

Fig. S7: ATR-FTIR spectrum of **C2**

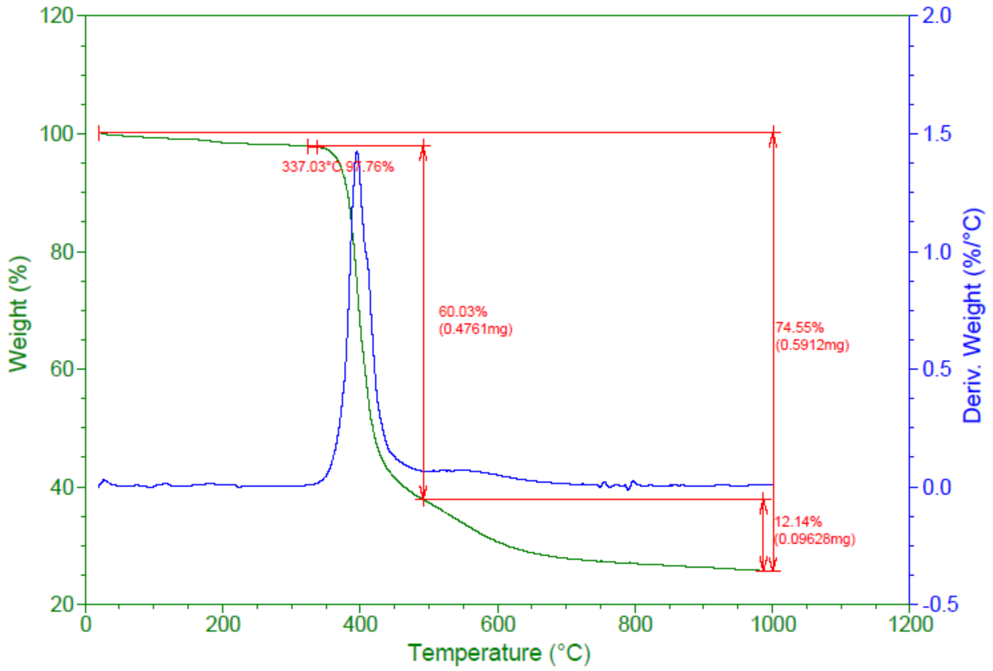

Fig. S8: TGA-DTG curves for **C2**

4. SCXRD studies

Diffraction data for **C1**-α crystal (colourless, prismatic, 0.18 x 0.25 x 0.37 mm^3) have been collected at ambient temperature by means of a Bruker-Axs three circle diffractometer equipped with the SMART-APEX CCD area detector and working with graphite-monochromated Mo-Kα X-radiation (λ = 0.7107 Å). Data reduction was performed with the SAINT software[3] and intensities were corrected for Lorentz and polarization effects.

Crystals of **C2**-β were selected and mounted on a MITIGEN holder in Paratone oil, then quickly placed in a nitrogen stream in order to extract the best data set avoiding the possible degradation upon desolvation or exposure to air. Diffraction data for **C2**-β crystal (colourless, prismatic, 0.12 x 0.28 x 0.35 mm^3) have been collected at 190 K by means of a Bruker-Nonius X8 four-circle diffractometer equipped with the APEX-II CCD area detector and working with graphite-monochromated Mo-Kα X-radiation (λ = 0.7107 Å). Data reduction, including intensity integrations and corrections for Lorentz and polarization effects, was performed with the APEX2 software.[4] Absorption effects for the diffraction data of both studied crystals were empirically evaluated by the SADABS software[5] and absorption corrections were applied to the data. The **C1**-α crystal structure was solved by direct methods (SIR 2019),[6] whereas the **C2**-β crystal structure was solved by dual-space iteration starting from Patterson superposition (SHELXT).[7] Both crystal structures were refined by full-matrix least-square procedures on F^2 using all reflections (SHELXL 2018/3)[8] and crystal data are reported in Table S1. Positions of hydrogens, belonging to a water solvent molecule in **C1**-α, were located in the final ΔF maps; these positions were refined in the final least-square refinement cycles, restraining the O-H distances to be 0.9 ± 0.01 Å. Unresolved positional disorder probably affected one of the two acetonitrile solvent molecules in the **C1**-α crystal, which resulted with large and elongated thermal ellipsoids. These atom sites were refined with soft restraints on the atom displacement parameters (ISOR and DELU) and on the molecular geometry (SAME). Positions of hydrogens belonging to water solvent molecules in the **C2**-β crystal remained undetermined and extensive positional disorder affected 5 of the 11 DMSO solvent molecules. Disorder resulted in apical S atoms occurring over two alternative positions, mutually exclusive and with the same statistical probability, which correspond to the two opposite vertexes of the trigonal pyramid having as a common base the two C and the O atoms. Atom belonging to DMSO solvent molecules were refined with soft restraints on the molecular geometries (SAME) and on the atom displacement parameters (ISOR, DELU and RIGU).

Crystals of **C2**-δ (~100 μm) were picked out of the liquor and immediately covered with immersion oil (Cargille, NVH) to avoid disintegration outside of the solvent. Single crystal data were collected, at room temperature, on the XRD2 beamline [9] at the Elettra synchrotron light-source (Trieste, Italy)

at a wavelength of 0.6199 Å. The light source obtained from the superconducting wiggler was monochromatized using a Si-crystal double monochromator. The diffraction setup consisted of an Arinax MD2S high throughput diffractometer and Pilatus 6M detector. A standard 360° φ-scan was used, with 1° step size. Data reduction, including intensity integrations and corrections for Lorentz and polarization effects, was performed using XDS.[10] The structure of **C2**-δ was solved using ShelXT and refined using ShelXL as implemented in the OLEX2 package.[11] Hydrogen atoms belonging to the organic moieties were placed at calculated positions with the appropriate AFIX instructions and refined using a riding model.

Considering the crystal structure without guest molecules, the free volume accessible to CO_2 were calculated (per unit cell) as solvent accessible surface for a spherical molecular probe of 1.72 Å radius and an approximate grid space of 0.7 Å. Calculations were carried out by the Mercury software[12].

Deposition Numbers 2172063, 2172064 and 2172328 (**C1**-α, **C2**-β and **C2**-δ, respectively) contain the supplementary crystallographic data for this paper. These data are provided free of charge by the joint Cambridge Crystallographic Data Centre and Fachinformationszentrum Karlsruhe Access Structures service.

Table S1. Single crystal data for the studied compounds.

	**C1**-α	**C2**-β	**C2**-δ
Formula	$C_{94}H_{86}N_{16}O_{13}$	$C_{130}H_{172}N_{12}O_{28}S_{11}$	$C_{72}H_{64}N_8O_8$
M	1647.79	2703.45	1169.31
crystal system	monoclinic	monoclinic	Triclinic
space group	$P2_1$ (no. 4)	$P2_1/n$ (no. 14)	$P\bar{1}$ (no. 2)
a (Å)	19.1383(13)	24.354(3)	14.423(3)
b (Å)	11.6804(8)	20.120(2)	25.413(5)
c (Å)	19.3415(14)	31.705(3)	32.957(7)
α (°)	90	90	85.77(3)
β (°)	92.2030(10)	109.434(5)	78.52(3)
γ (°)	90	90	78.30(3)
V (Å^3)	4320.5(5)	14650(3)	11585(4)
Z	2	4	6
ρ_{calcd} (g cm^{-3})	1.267	1.226	1.006
Wavelength, λ (Å)	Mo-Kα	Mo-Kα	0.6199
μ at λ (mm^{-1})	0.087	0.235	0.050
min/max transmission	0.876/0.985	0.896/0.978	

θ range (°)	2.04-25.06	2.52-25.00	1.43-64.26
measured reflections	43404	238616	270908
unique reflections	15199	25747	80293
R_{int}	0.041	0.119	0.2606
strong data [$I_O>2\sigma(I_O)$]	9750	12991	14895
refined parameters	1116	1680	2390
$R1$, w$R2$ strong data	0.0464, 0.1056	0.1364, 0.3732	0.1070, 0.2863
$R1$ w$R2$ all data	0.0840, 0.1243	0.2180, 0.4198	0.2932, 0.3913
max/min residual (eÅ^{-3})	0.28/-0.20	1.23/-0.91	0.35/-0.28

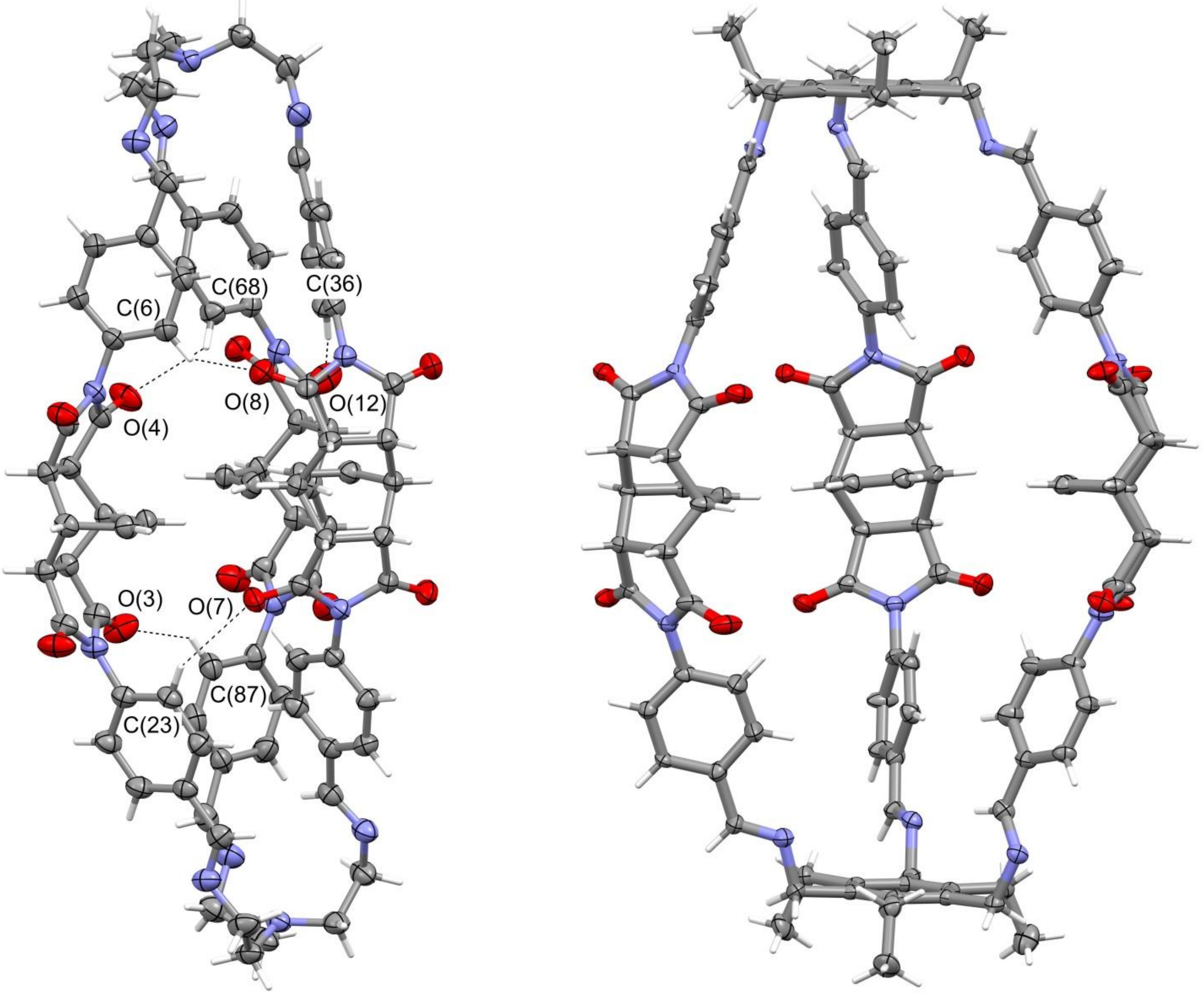

Fig. S9. Plot showing thermal ellipsoids for the **C1**-α (left) and **C2**-β (right) molecular cages (ellipsoids are drawn at the 30% probability level; additional water, MeCN and DMSO solvent molecules are omitted for clarity). The central cavity of **C1** is collapsed and this conformation is stabilized by weak intramolecular C_{aryl}-H⋯O H-bonds (drawn ad dashed lines) whose geometrical features are: C(6)⋯O(8) 3.33(1) Å, H(6)⋯O(8) 2.71(1) Å, C(6)-H(6)⋯O(8) 124.7(3)°; C(23)⋯O(7) 3.49(1) Å, H(23)⋯O(7) 2.64(1) Å, C(23)-H(23)⋯O(7)

152.3(3)°; C(36)⋯O(12) 3.22(1) Å, H(36)⋯O(12) 2.40(1) Å, C(36)-H(36)⋯O(12) 147.4(4)°; C(68)⋯O(4) 3.17(1) Å, H(68)⋯O(4) 2.45(1) Å, C(68)-H(68)⋯O(4) 134.4(3)°; C(87)⋯O(3) 3.16(1) Å, H(87)⋯O(3) 2.42(1) Å, C(87)-H(87)⋯O(3) 136.8(3)°.

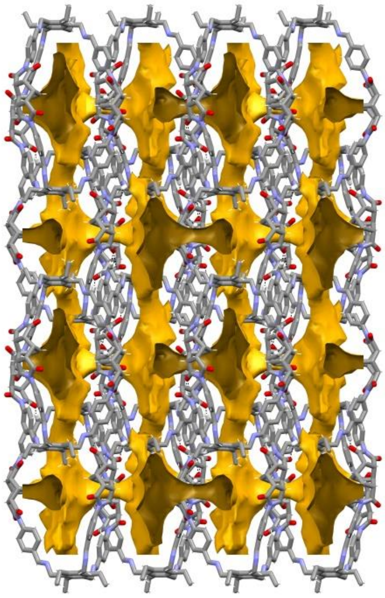

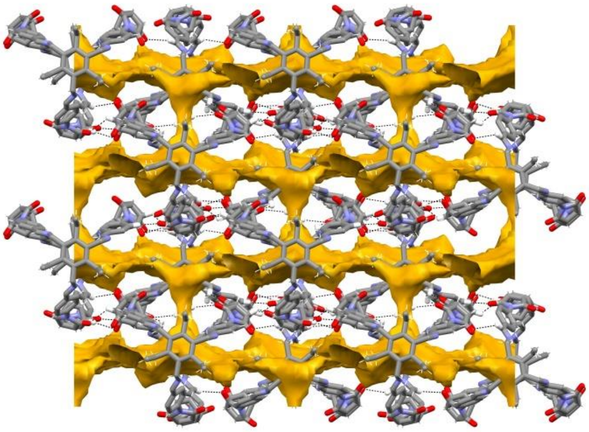

Fig. S10: from top to bottom, views of the packing along **a** and **c** axes for the **C2-**β cage. Solvent molecules (DMSO and water) have been omitted from the calculations. The empty spaces, calculated as solvent accessible surface for a molecular probe of 1.72 Å radius, are shown in yellow, while H-bonding interactions are drawn as dashed lines.

Table S2. Geometrical features of the weak intermolecular C-H···O hydrogen bonds connecting adjacent molecular cages in the crystals of **C1**-α and **C2**-β.

C1-α

Donor group	D···A (Å)	H···A (Å)	D-H···A (°)	Acceptor atom
C_{sp3}(1)-H(1B)	3.33(1)	2.66(1)	126.2(3)	O(8)^{1}
C_{sp3}(11)-H(11)	3.53(1)	2.68(1)	145.8(3)	O(7)^{2}
C_{sp3}(18)-H(18)	3.09(1)	2.54(1)	115.5(3)	O(2)^{2}
C_{sp3}(42)-H(42)	3.06(1)	2.46(1)	119.1(3)	O(9)^{3}
C_{sp3}(43)-H(43)	3.18(1)	2.64(1)	114.7(3)	O(9)^{6}
C_{sp3}(72)-H(72)	3.46(1)	2.54(1)	156.5(3)	O(6)^{4}
C_{sp3}(73)-H(73)	3.31(1)	2.63(1)	127.1(3)	O(11)^{5}
C_{sp3}(76)-H(76)	3.12(1)	2.60(1)	113.3(3)	O(6)^{5}
C_{aryl}(83)-H(83)	3.08(1)	2.46(1)	123.9(3)	O(9)^{6}

C2-β

Donor group	D···A (Å)	H···A (Å)	D-H···A (°)	Acceptor atom
C_{sp2}(8)-H(8)	3.43(1)	2.63(1)	142.1(4)	O(7)^{7}
C_{sp3}(17)-H(17)	3.54(1)	2.54(1)	171.0(4)	O(8)^{8}
C_{sp2}(33)-H(33)	3.28(1)	2.61(1)	128.1(4)	O(9)^{9}
C_{sp3}(37)-H(37B)	3.31(1)	2.67(1)	122.9(4)	O(12)^{10}
C_{aryl}(43)-H(43)	3.33(1)	2.42(1)	158.9(5)	O(2)^{11}
C_{sp3}(53)-H(53)	3.36(1)	2.72(1)	122.0(4)	O(1)^{11}
C_{sp3}(64)-H(64A)	3.15(1)	2.55(1)	118.7(4)	O(11)^{12}
C_{sp3}(76)-H(76)	3.32(1)	2.57(1)	131.1(4)	O(3)^{13}
C_{sp3}(78)-H(78)	3.25(1)	2.49(1)	132.4(4)	O(3)^{13}
C_{sp3}(106)-H(06C)	3.65(1)	2.68(1)	171.1(6)	O(11)^{12}

Symmetry code: (^{1}) = -*x*+1, *y*-$^{1}/_{2}$, -*z*+2; (^{2}) = -*x*+1, *y*-$^{1}/_{2}$, -*z*+1; (^{3}) = *x*, *y*+1, *z*; (^{4}) = *x*, *y*-1, *z*; (^{5}) = -*x*, *y*-$^{1}/_{2}$, -*z*+1; (^{6}) = -*x*, y+$^{1}/_{2}$, -*z*+1; (^{7}) = *x*-$^{1}/_{2}$, -*y*+$^{1}/_{2}$, *z*-1/2; (^{8}) = -*x*+$^{3}/_{2}$, *y*-$^{1}/_{2}$, -*z*+$^{3}/_{2}$; (^{9}) = *x*+$^{1}/_{2}$, -*y*+$^{1}/_{2}$, *z*+$^{1}/_{2}$; (^{10}) = -*x*+1, -*y*+1, -*z*+1; (^{11}) = -*x*+$^{3}/_{2}$, *y*+$^{1}/_{2}$, -*z*+$^{3}/_{2}$; (^{12}) = -*x*+1, -*y*+1, -*z*+2; (^{13}) = -*x*+$^{1}/_{2}$, *y*+$^{1}/_{2}$, -*z*+$^{3}/_{2}$.

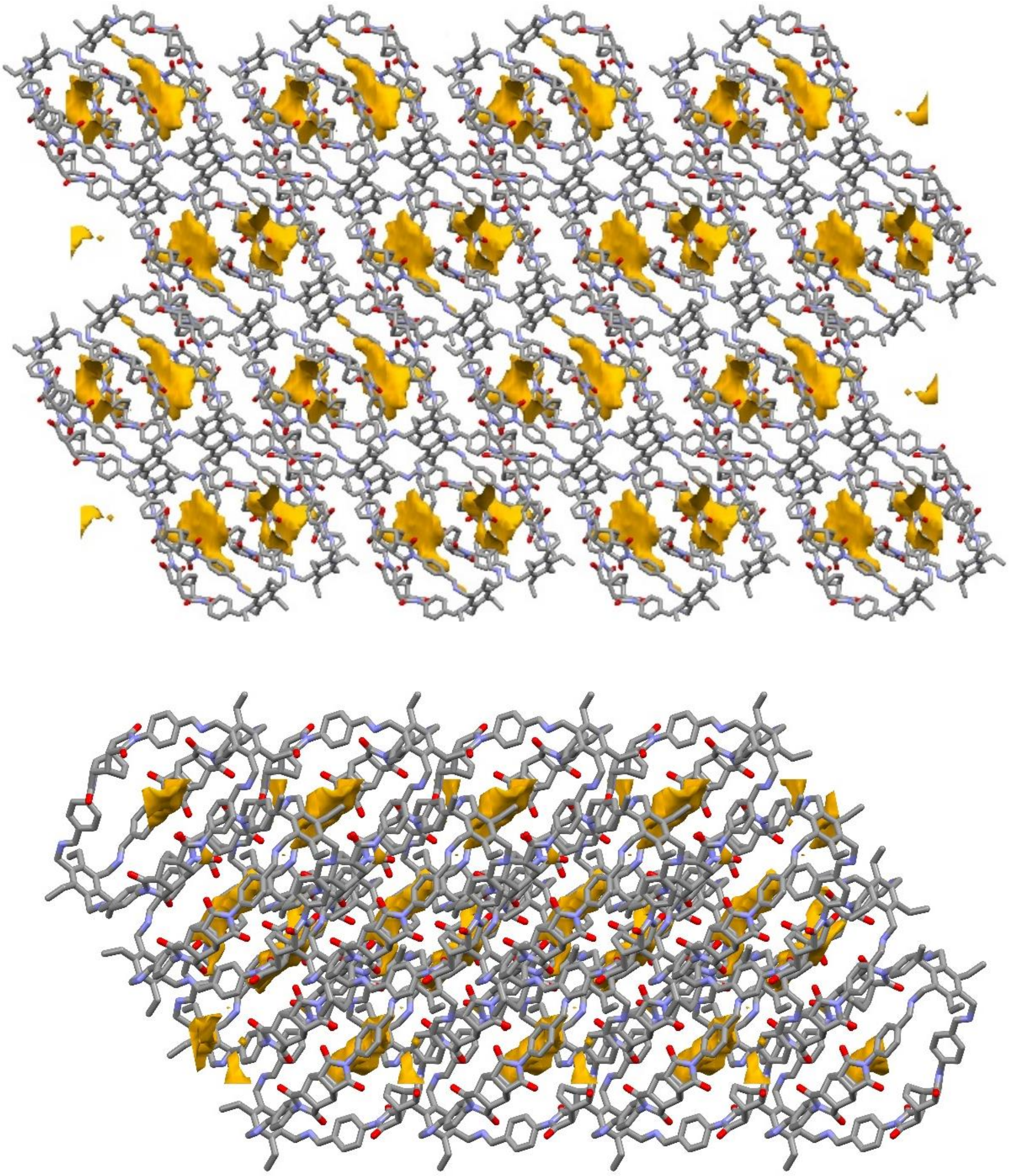

Fig. S11: from top to bottom, views of the packing along **a** and **c** axes for the **C2-**δ cage. Solvent molecules (MeCN) have been omitted from the calculations. The empty spaces, calculated as solvent accessible surface for a molecular probe of 1.72 Å radius, are shown in yellow.

5. Gas adsorption studies on C1 and C2

5.1 Nitrogen adsorption studies at 77 K

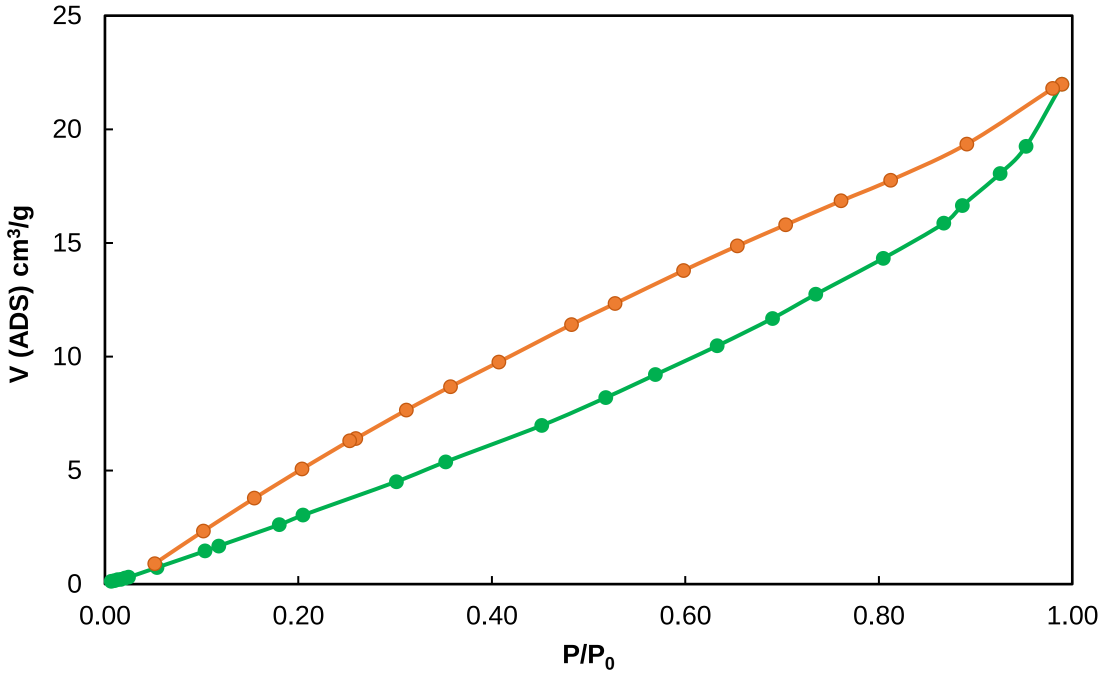

Fig. S12: N_2 isotherm measured for **C1** at 77 K (green line: adsorption; orange line: desorption; dots: experimental data).

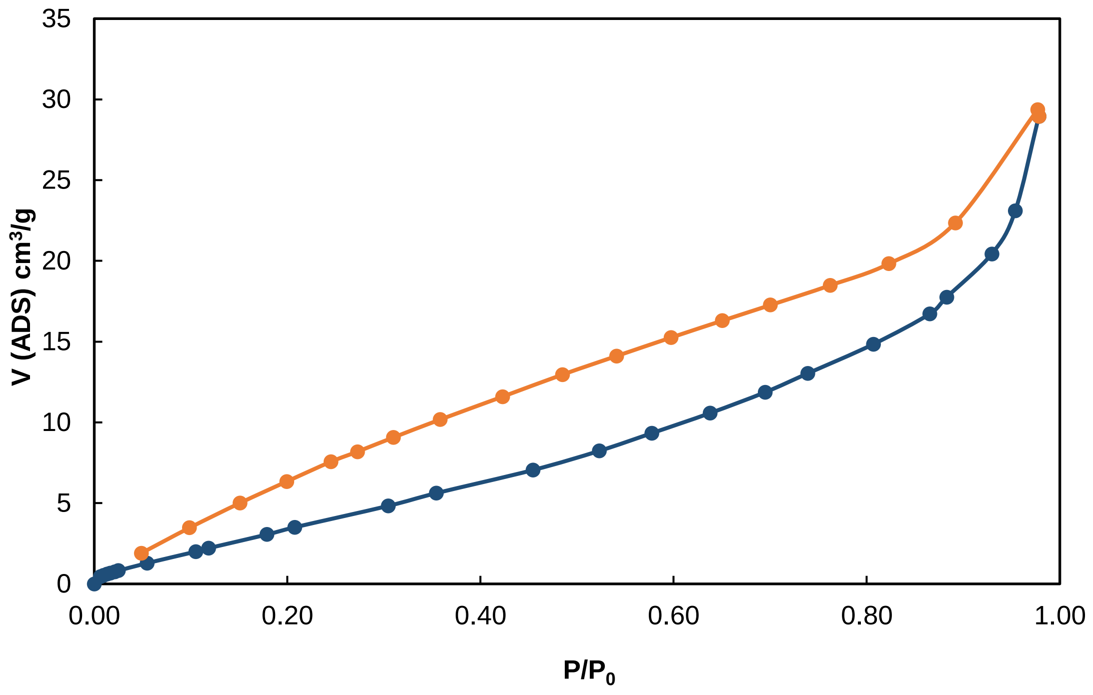

Fig. S13: N_2 isotherm measured for **C2** at 77 K (blue line: adsorption; orange line: desorption; dots: experimental data).

5.2 Nitrogen adsorption studies at 298 K

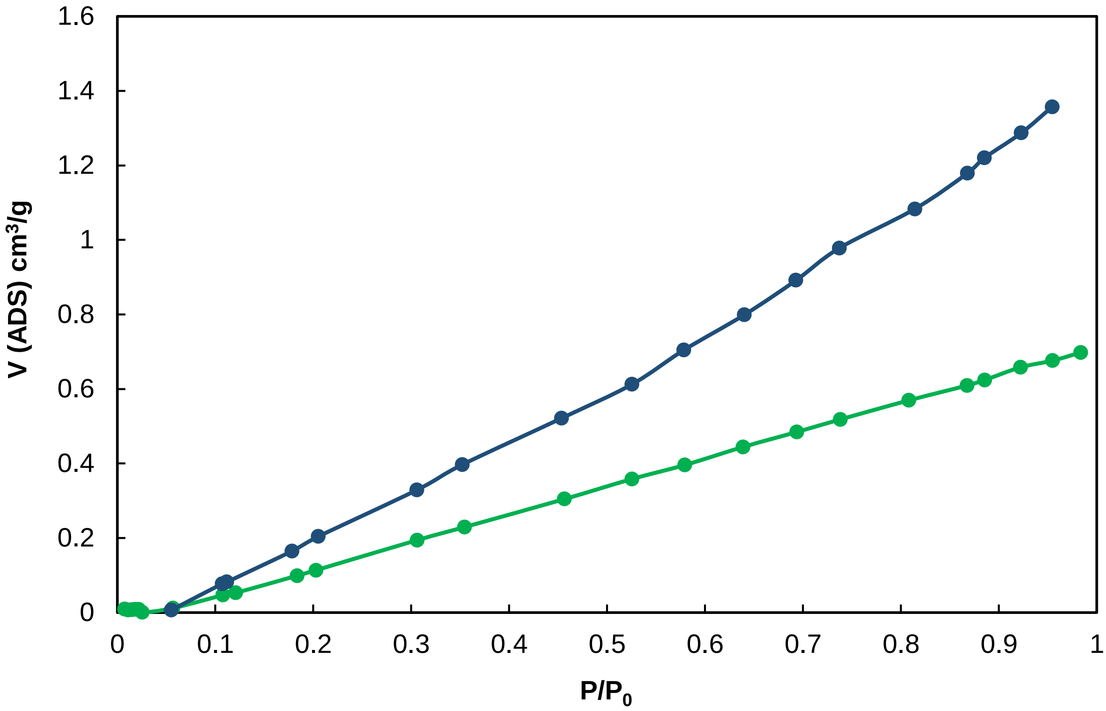

Fig. S14: Overlay of N_2 adsorption isotherms, measured for **C1** (blue) and **C2** (green) at 298 K. P_0 = 1 bar, which is the maximum pressure reached by the instrument.

5.3 CO_2 adsorption studies

5.3.1 CO_2 uptake

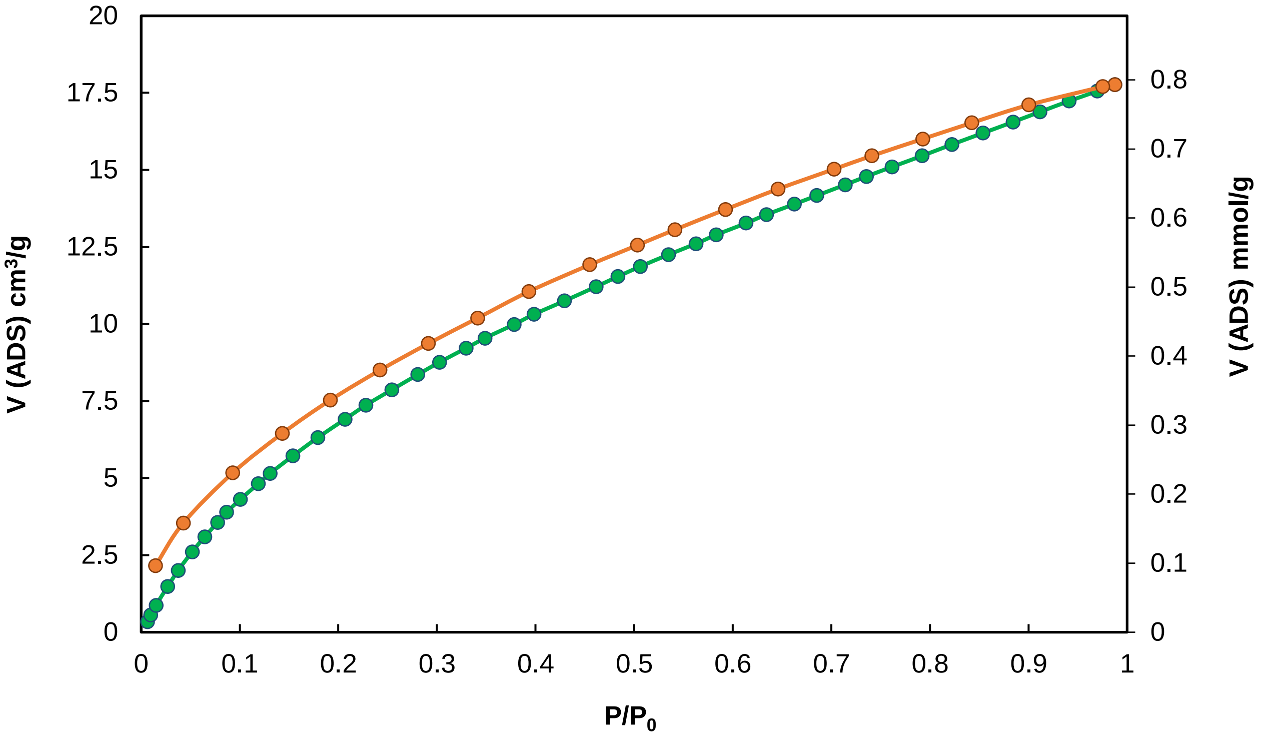

Fig. S15: CO_2 isotherm measured for **C1** at 273 K (green line: adsorption; orange line: desorption; dots: experimental data). P_0 = 1 bar, which is the maximum pressure reached by the instrument.

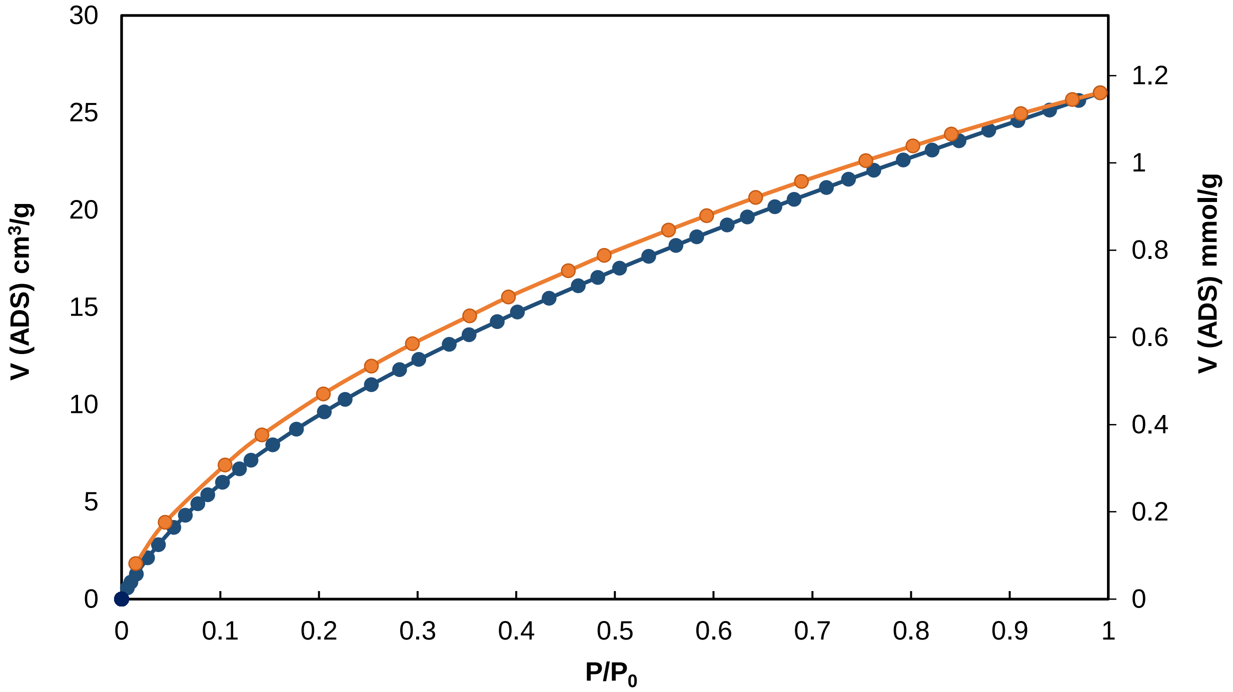

Fig. S16: CO_2 isotherm measured for **C2** at 273 K (blue line: adsorption; orange line: desorption; dots: experimental data). P_0 = 1 bar, which is the maximum pressure reached by the instrument.

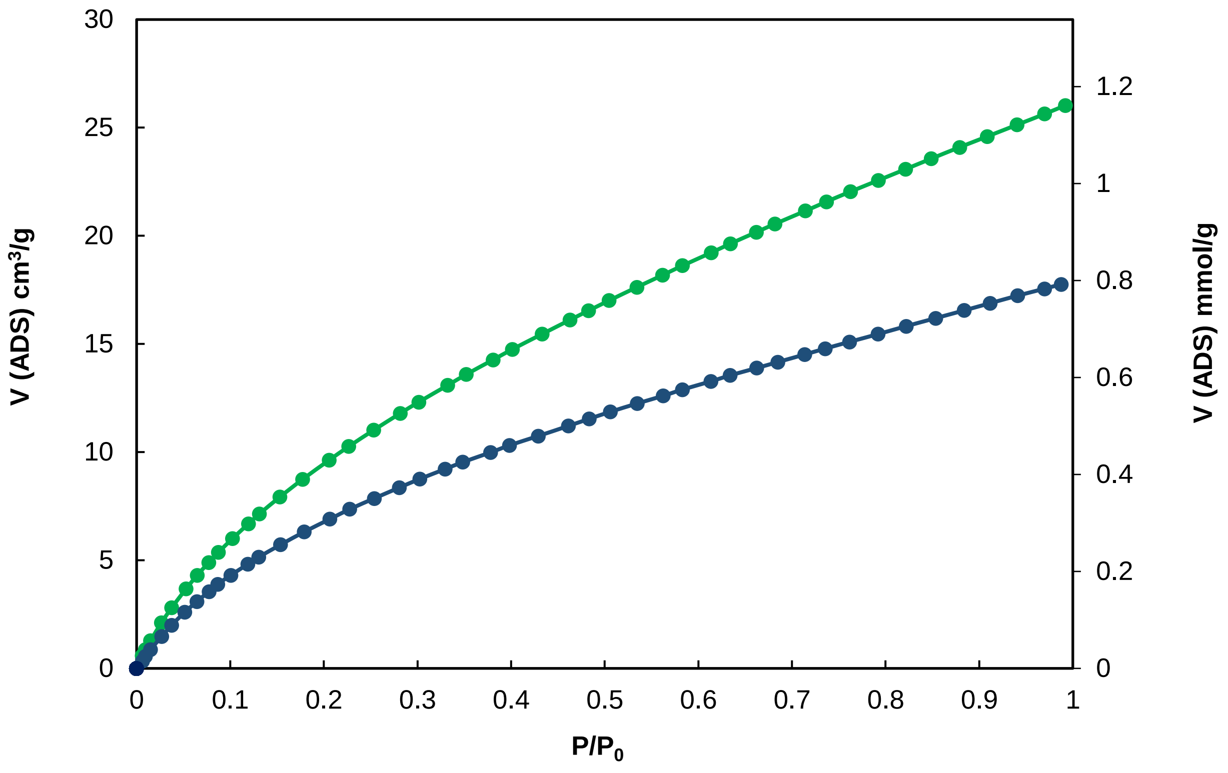

Fig. S17: Overlay of CO_2 adsorption isotherms, measured for **C1** (blue) and **C2** (green) at 273 K. P_0 = 1 bar, which is the maximum pressure reached by the instrument.

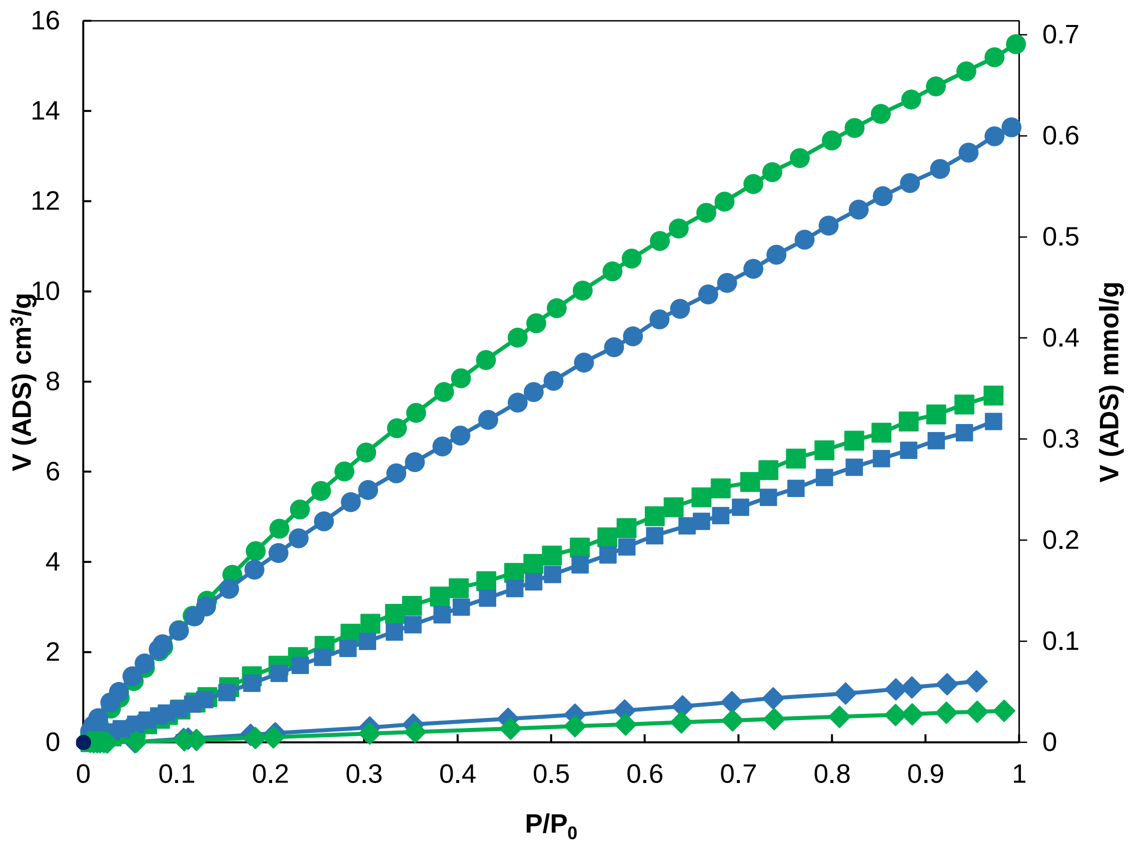

Fig. S18: Overlay of CO_2 (circles), CH_4 (squares), and N_2 (diamonds) adsorption isotherms, measured for **C1** (blue) and **C2** (green) at 298 K. P_0 = 1 bar, which is the maximum pressure reached by the instrument.

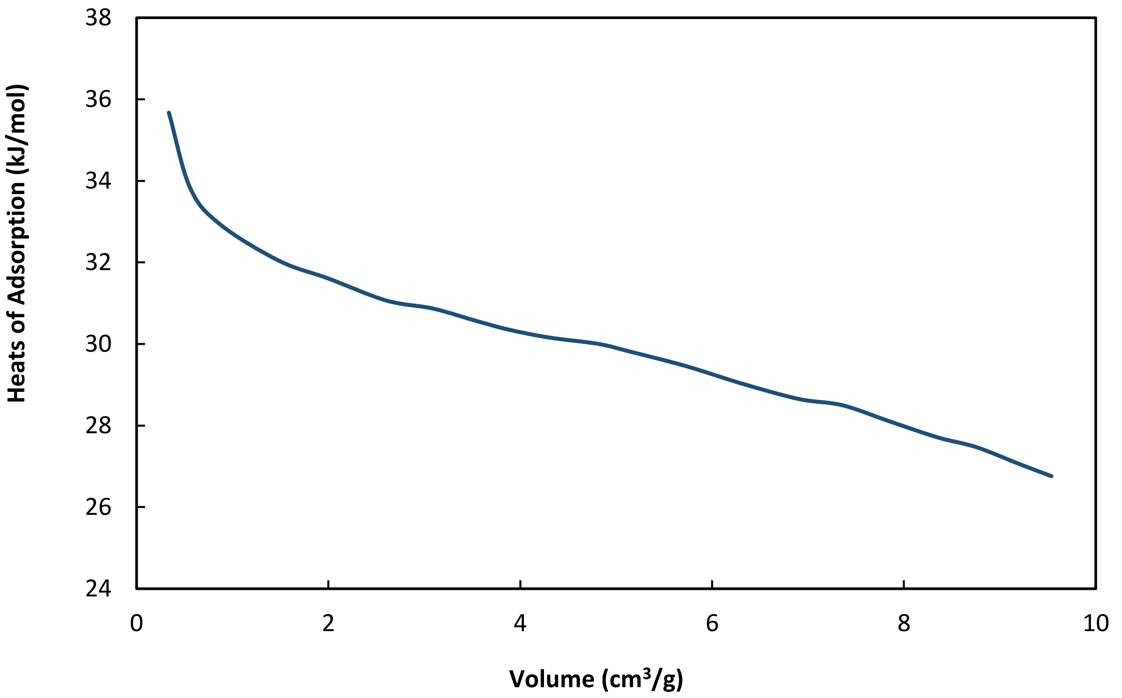

Fig. S19: Heats of adsorption for **C1**

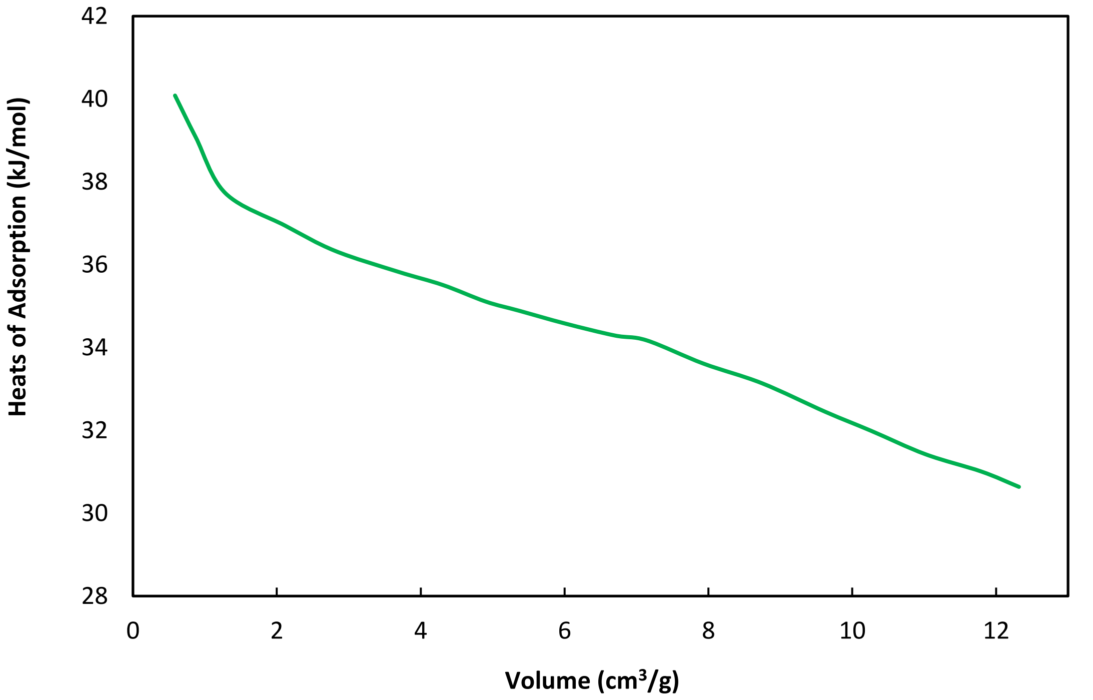

Fig. S20: Heats of adsorption for **C2**.

5.3.2 Pore size distribution (PSD)

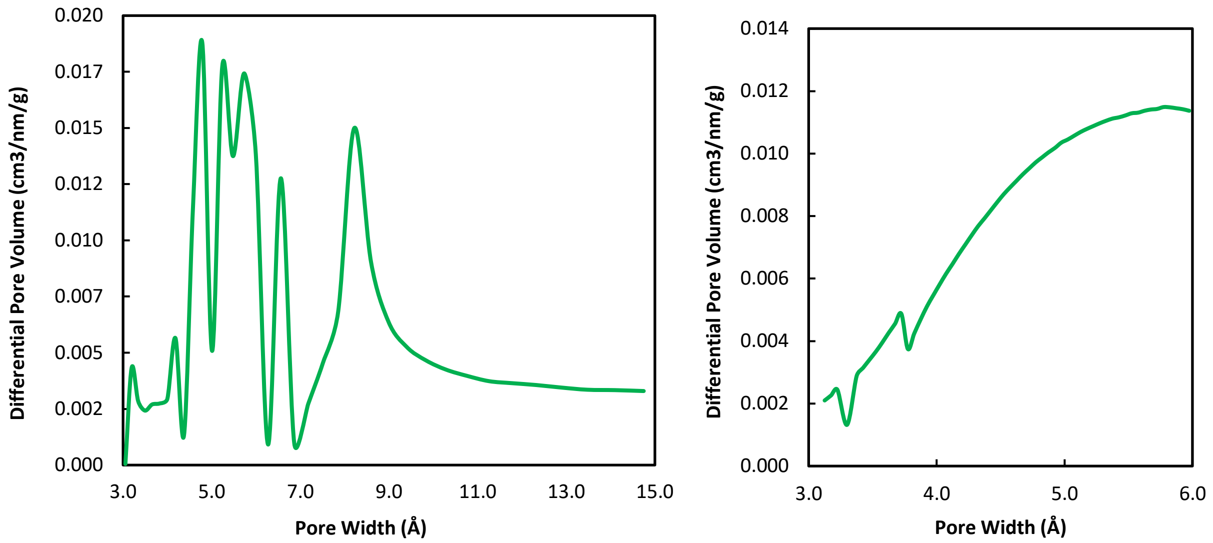

Fig. S21: NLDFT (left) and H-K (right) pore size distribution for **C1** from CO_2 at 273 K

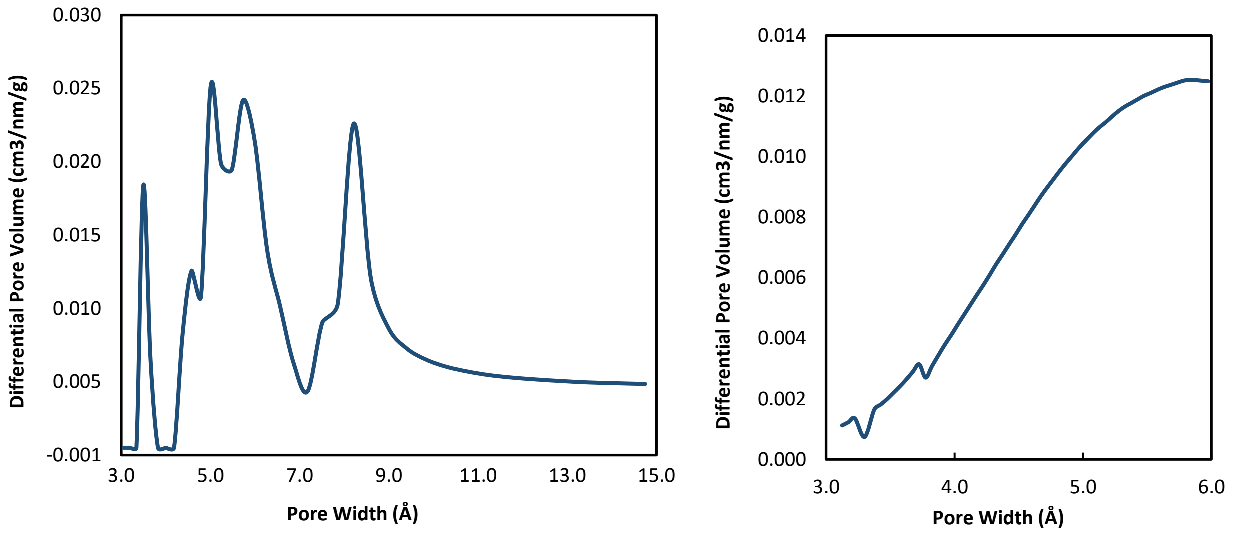

Fig. S22: NLDFT (left) and H-K (right) pore size distribution for **C2** from CO_2 at 273 K

5.3.3 Ideal selectivity for potential post-combustion applications (IAST)

The ideal adsorption solution theory (IAST), reported for the first time by Myers and Prausnitz, is a method created to predict the selectivity of binary gas mixtures by the simple analysis of single gas adsorption isotherms.[13] It is currently used to predict the selectivity for porous materials and it is found to be fairly accurate.[14] To assess the potential use of the material for post-combustion

applications from flue-gas streams (CO_2/N_2 separation), the curves obtained from CO_2 and N_2 adsorption measurements at 298 K were fitted with dual site Langmuir-Freundlich equation using the IAST++ software[15] and the ideal selectivity was estimated for a potential CO_2/N_2 mixture (typically 15% CO_2 and 85% N_2 for flue gas) and calculated according to the formula:

$$S = \frac{Q_{CO2}}{Q_{N2}} \times \frac{P_{N2}}{P_{CO2}}$$

Where:

- P_{CO2} is the partial pressure of CO_2
- P_{N2} is the partial pressure of N_2
- Q_{N2} is the N_2 uptake
- Q_{CO2} is theCO_2 uptake

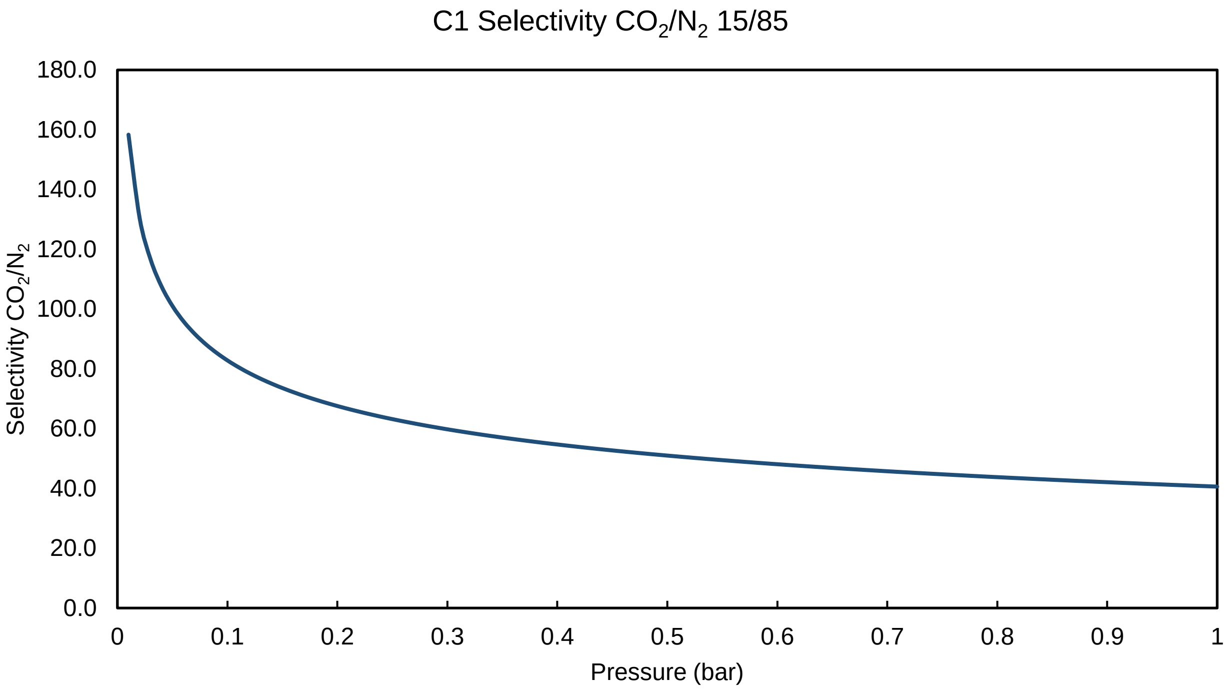

Fig. S23: Ideal CO_2/N_2 selectivity for **C1** for a potential 15/85 mixture

.

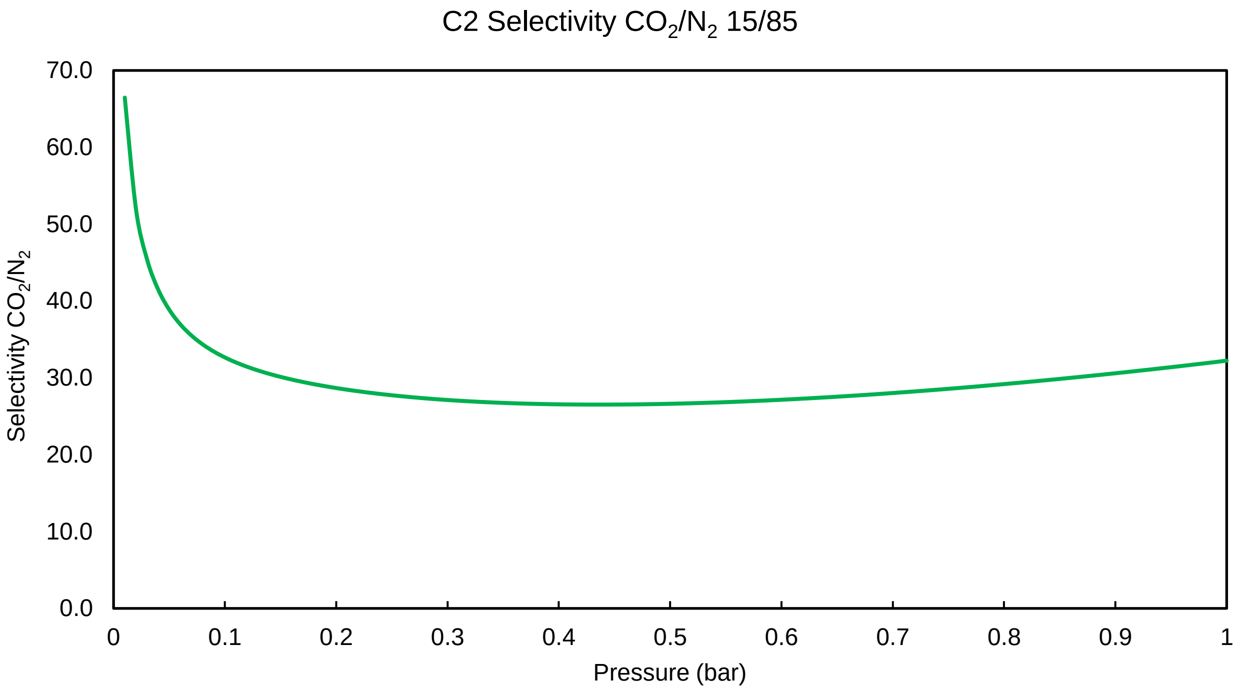

Fig. S24: Ideal CO_2/N_2 selectivity for **C2** for a potential 15/85 mixture

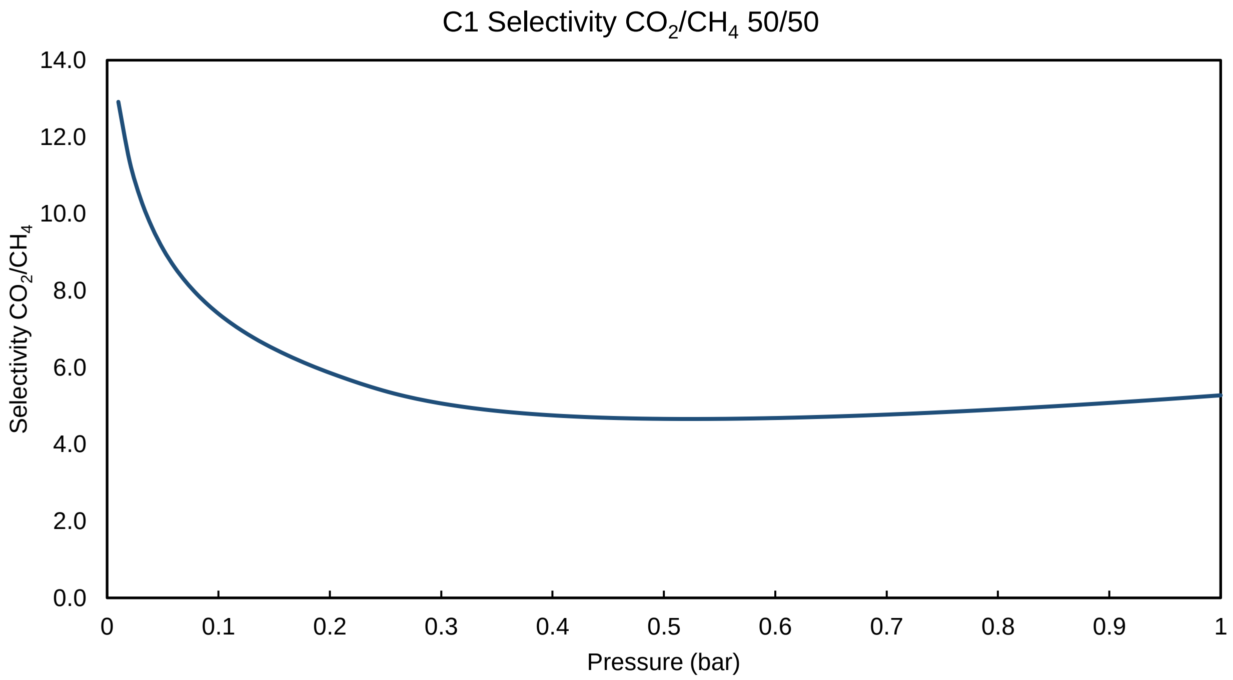

Fig. S25: Ideal CO_2/CH_4 selectivity for **C1** for a potential 50/50 mixture

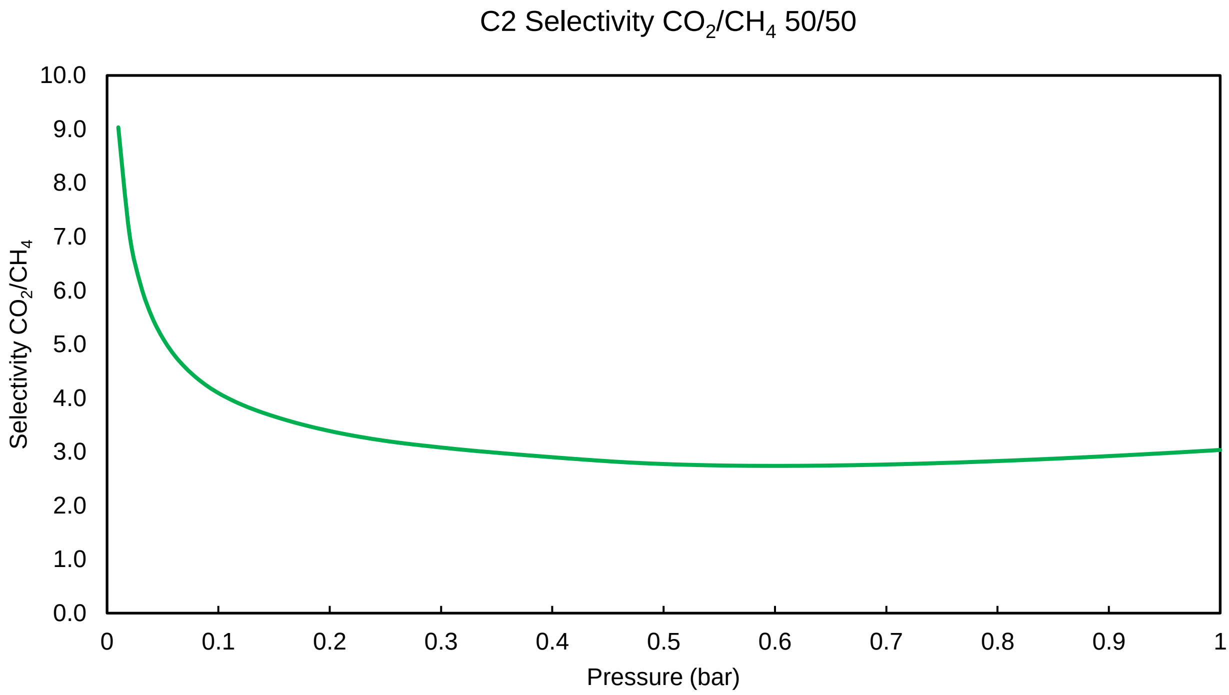

Fig. S26: Ideal CO_2/CH_4 selectivity for **C2** for a potential 50/50 mixture

5.4 The summary of gas adsorption for organic cages reported in the literature

Cage	State[a]	SA_{BET}[b] m^2/g	CO_2 cm^3/g 1 bar, 298K	N_2 cm^3/g 1 bar, 298K	CH_4 cm^3/g 1 bar, 298K	CO_2/N_2 selectivity 1 bar, 298K		CO_2/CH_4 IAST selectivity	Q_{st} CO_2 kJ/mol[f]	Ref
						IAST[d]	Henry[e]			
**C1** 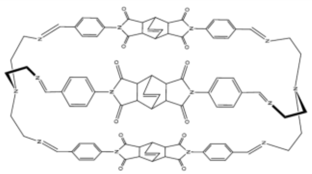	**A**	**44 (224 CO_2[c])**	**13.6 (17.9@273K)**	**1.36**	**7.1**	**41**	**-**	**5.3**	**35**	
**C2** 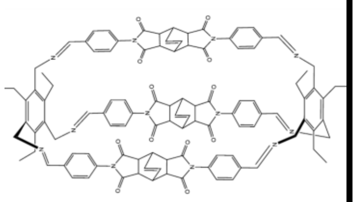	**MC**	**35 (325 CO_2[c])**	**15.5 (26@273K)**	**0.67**	**7.7**	**32**	**-**	**3.0**	**39**	
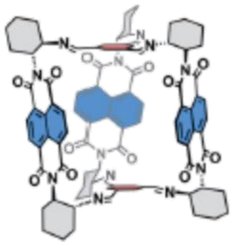	C	522	41.2 (65.2@273K)	4.26 (6.94@273K)	15 (24.4@273K)	17.8 (26.9@273K)	-		27.0	16

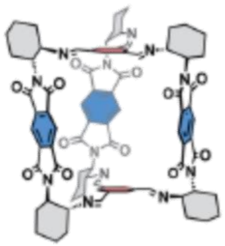	C	-	28.2 (44.8@273K)	18.6 (3.58@273K)	8.1 (11.2@273K)	28.0 (45.5@273K)	-		27.5	16
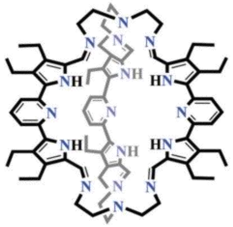	C	279 (CO_2)[c]	96 (196K)	16 (77K)	29 (196K)	-	-		-	17
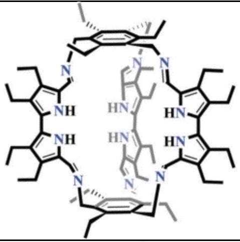	C	111 (CO_2)[c]	51 (196K)	1 (77K)	8 (196K)	-	-		-	17
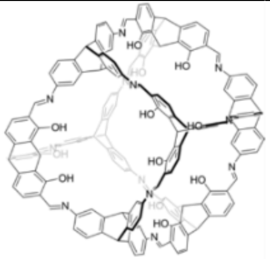	A	1014	92 (273K)	10 (273K)	29.1 (273K)	-	33.7 (273K)	7.67[e] (273K)	24.2	18
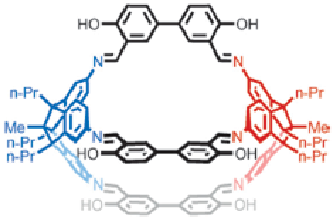	A	211-918	56–78 (273K)	7.8–9.6 (273K)	22–28 (273K)	-	17.5–25.3 (273K)	3.75-4.93[e] (273K)	25-31	19

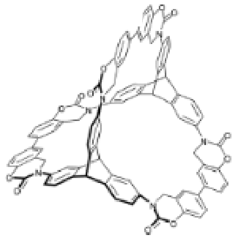	MC/A	105	58.8 (273K)	14.5 (273K)	21.8 (273K)	-	13.9 (273K)	-	60.1	20

- n.a. = not available; [a] A = amorphous, C = crystals, MC = microcrystals; P = polymorph; [b] = N_2, 77 K, 1 bar; [c] = CO_2, 273 K, 1 bar; [d] = IAST selectivity at 1 bar; [e] = calculated from Henry law costants [18], [f] z. c. = zero coverage [18]

Table S3: **C1**, **C2** and examples of organic cages taken from the literature.

6. Single gas permeation studies on mixed matrix membranes

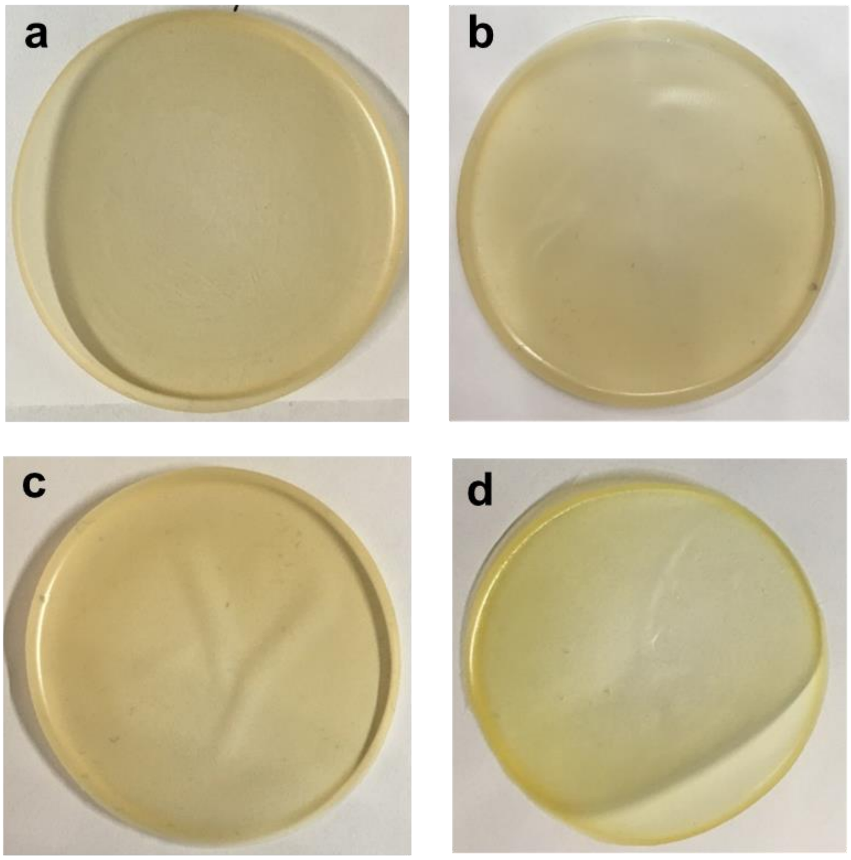

Fig. S27: Optical photographs of the mixed matrix membranes studied in this work. a) PEEK-WC/**C1**; b) PEEK-WC/**C2**; c) Matrimid®9725/**C1**; d) Matrimid®9725/**C2.**

6.1 Theory[2]

According to the solution–diffusion model, the permeation occurs in three steps: i) gas molecules dissolve in the membrane on the feed side, ii) gas molecules diffuse through the membrane, from the feed side to the permeate side, iii) the gas molecules desorb from the permeate side into the permeate reservoir. The diffusion process is the rate-determining step, depending i) on the size of the penetrating gas molecules, ii) on the space among polymer chains (free volume) and on their rigidity. The permeability (*P*) of a certain gas can be expressed as the product between diffusivity (*D*) and solubility (*S*):

$$P = D \times S \qquad \text{(Eq. 1)}$$

Permeability expresses the ability of the membrane to be permeated by a certain gas. Its value represents the flux of permeate generated by the difference of pressure between the two sides of the

membrane, normalized for its surface area and thickness, and it is commonly expressed in Barrer (1 Barrer = 10^{-10} cm^3_{STP} cm cm^{-2} s^{-1} $cmHg^{-1}$).

For a pair of gases, the selectivity ($\alpha_{a,b}$) is determined by the ratio of the permeability of gas *a* and the permeability of gas *b*:

$$\alpha_{a,b} = \frac{P_a}{P_b} \qquad \text{(Eq. 2)}$$

In mixed matrix membranes, the filler influences the transport properties with respect to the pure polymer. One of the most applied model - employed in the description of this effect - is the Maxwell model[21,22], valid for spherical fillers up to approximately 30 vol.% concentration in the membrane. The MMM permeability, P_{MMM}, is given by:

$$P_{MMM} = P_C \left[\frac{P_d + 2P_c - 2\Phi_d\,(P_c - P_d)}{P_d + 2P_c + \Phi_d\,(P_c - P_d)}\right] \qquad \text{(Eq. 3)}$$

where P_c and P_d are the permeability of the continuous and dispersed phase, respectively, while Φ_d is the volume fraction of the dispersed phase. Thus, if the dispersed phase is more permeable than the continuous phase, the overall permeability increases, with a maximum theoretical limit given by $P_d = \infty$:

$$P_{MMM,max} = P_C \left[\frac{1 + 2\Phi_d}{1 - \Phi_d}\right] \qquad \text{(Eq. 4)}$$

The overall permeability decreases if the permeability of the dispersed phase is lower than that of the bulk polymer, with a minimum limit for $P_d = 0$:

$$P_{MMM,min} = P_C \left[\frac{1 - \Phi_d}{1 + 0.5\Phi_d}\right] \qquad \text{(Eq. 5)}$$

6.2 Experimental measurements

Single gas permeation studies were performed on circular membranes (exposed area 13.84 cm^2) at 25 °C and at a feed pressure of 1 bar by a fixed volume/pressure increase instrument designed by HZG and constructed by EESR (Geesthacht, Germany). Further details on the measurement protocols and data treatment are described in a previous paper.[23] Before each measurement, the membranes were evacuated in the testing cell by a turbo-molecular pump until complete desorption of all previously adsorbed gases and humidity. For the same reason, between two consecutive tests, the membranes were evacuated for a time equal to 10 times the time lag of the previous gas.

The time lag method was used for the determination of the permeability (P), diffusion (D), and solubility coefficients (S), which can be obtained from the increase of the permeate pressure, p_t, as a function of time, t, after exposure of the membrane to the gas[24]:

$$p_t = p_0 + \left(\frac{dp}{dt}\right)_0 \cdot t + \frac{RT}{V_P \cdot V_m} \cdot A \cdot l \cdot p_f \cdot S \times \left(\frac{D \cdot t}{l^2} - \frac{1}{6} - \frac{2}{\pi^2} \sum_{n=1}^{\infty} \frac{(-1)^n}{n^2} \exp\left(-\frac{D \cdot n^2 \cdot \pi^2 \cdot t}{l^2}\right)\right) \quad \text{(Eq. 6)}$$

where p_0 and $(dp/dt)_0$ are the starting pressure and baseline slope, respectively, which should be negligible in a well-evacuated and leak free membrane and permeability instrument. R is the universal gas constant, T the absolute temperature, V_P the permeate volume, V_m the molar volume of a gas at standard temperature and pressure [22.41 × 10^3 $m^{-3}{}_{STP}mol^{-1}$ at 0 °C and 1 atm], A the exposed membrane area, l its thickness, p_f the feed pressure, S the gas solubility and D the diffusion coefficient. The permeability P was obtained from the permeation curve (Eq. 7) in the pseudo steady-state:

$$P_t = P_0 + \left(\frac{dp}{dt}\right)_0 . t + \frac{RT\,A}{V_p\,V_m} . \frac{p_f\,p}{l} \left(t - \frac{l^2}{6D}\right) \quad \text{(Eq. 7)}$$

The diffusion coefficient is inversely proportional to time lag (Θ) and was calculated from (Eq. 8):

$$\Theta = \frac{l^2}{6D} \quad \text{(Eq. 8)}$$

The solubility coefficient (S), was calculated from the solution–diffusion transport model (Eq. 9):

$$S = P/D \quad \text{(Eq. 9)}$$

7. NMR and mass spectra of new compounds

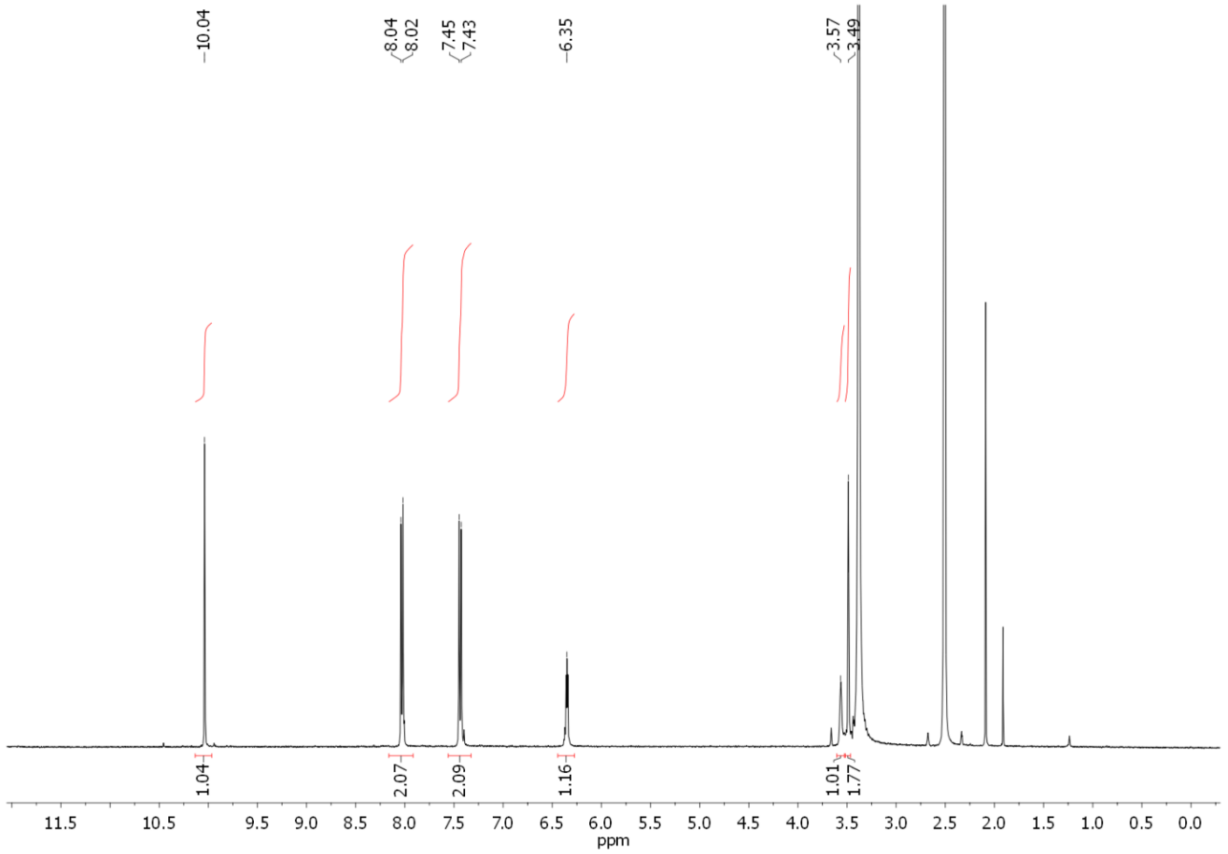

Fig. S28: ^{1}H-NMR spectrum (400 MHz; d^6-DMSO) of **1**

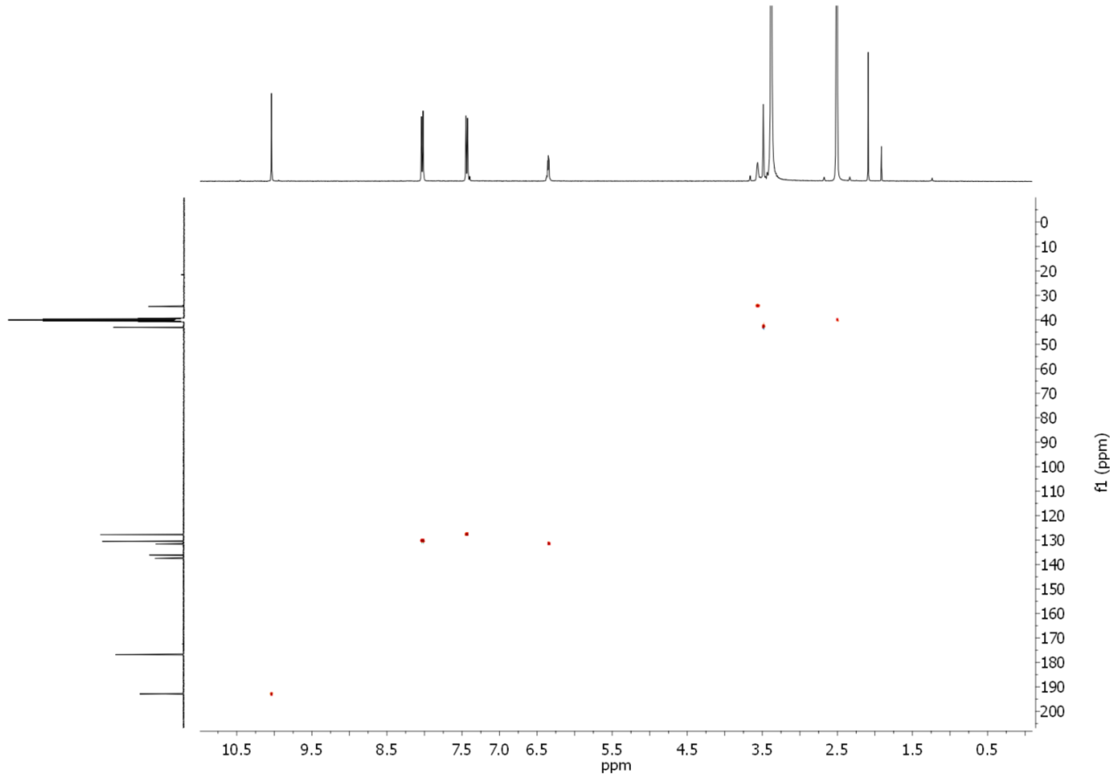

Fig. S29: HSQC spectrum (d^6-DMSO) of **1**

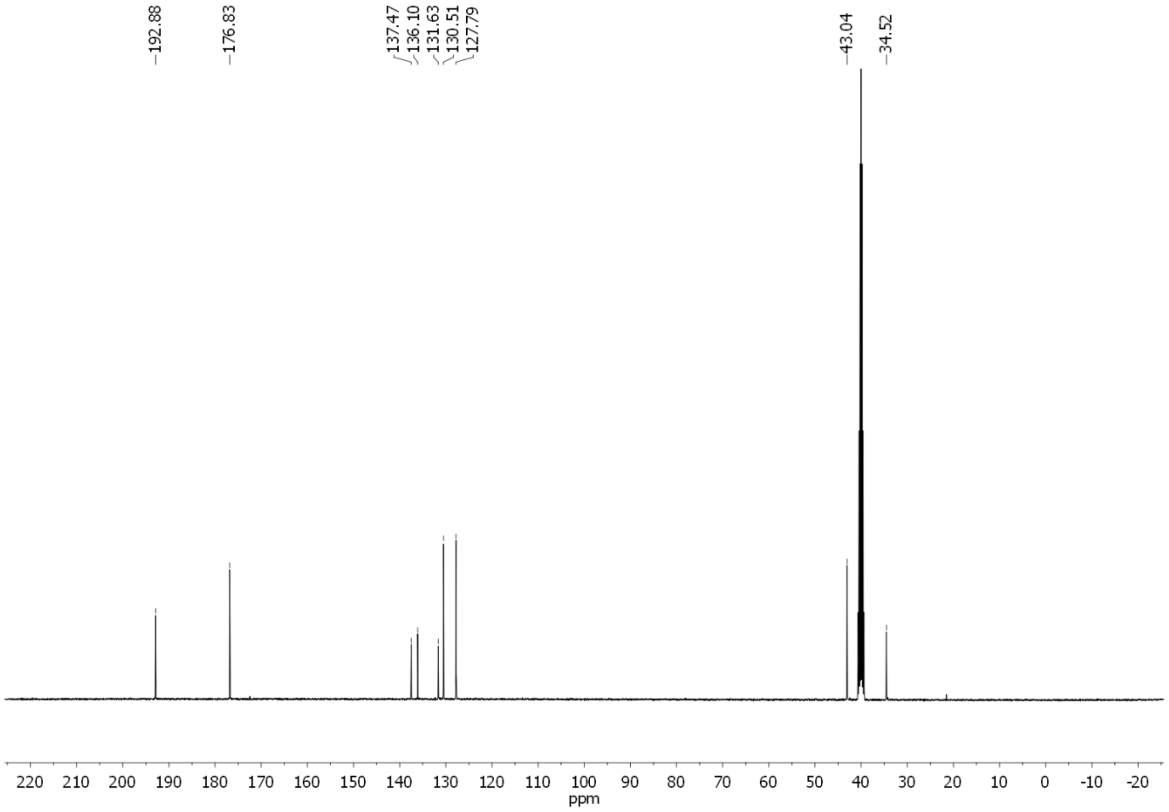

Fig. S30: ^{13}C-NMR spectrum (100 MHz; d^6-DMSO) of **1**

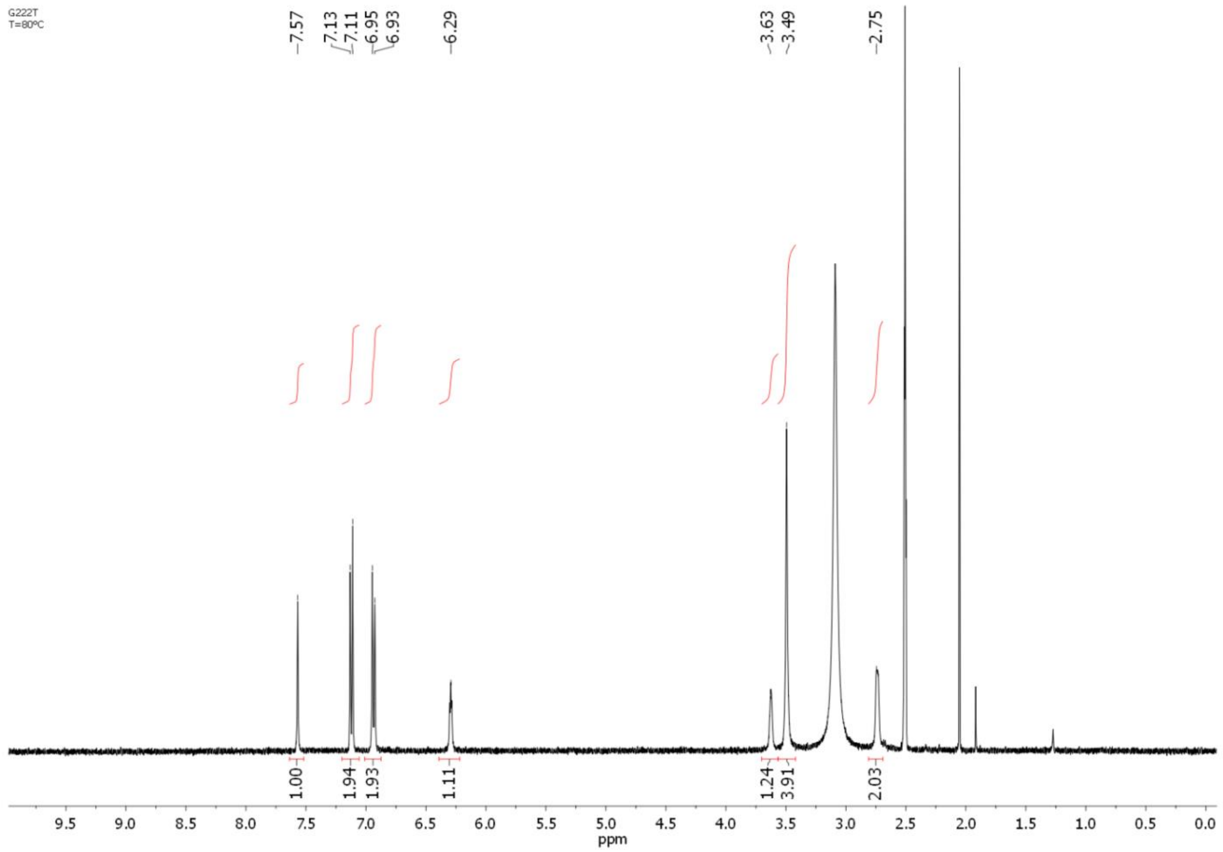

Fig. S31: ^1H-NMR(400 MHz; d^6-DMSO) of **C1** at 80 °C

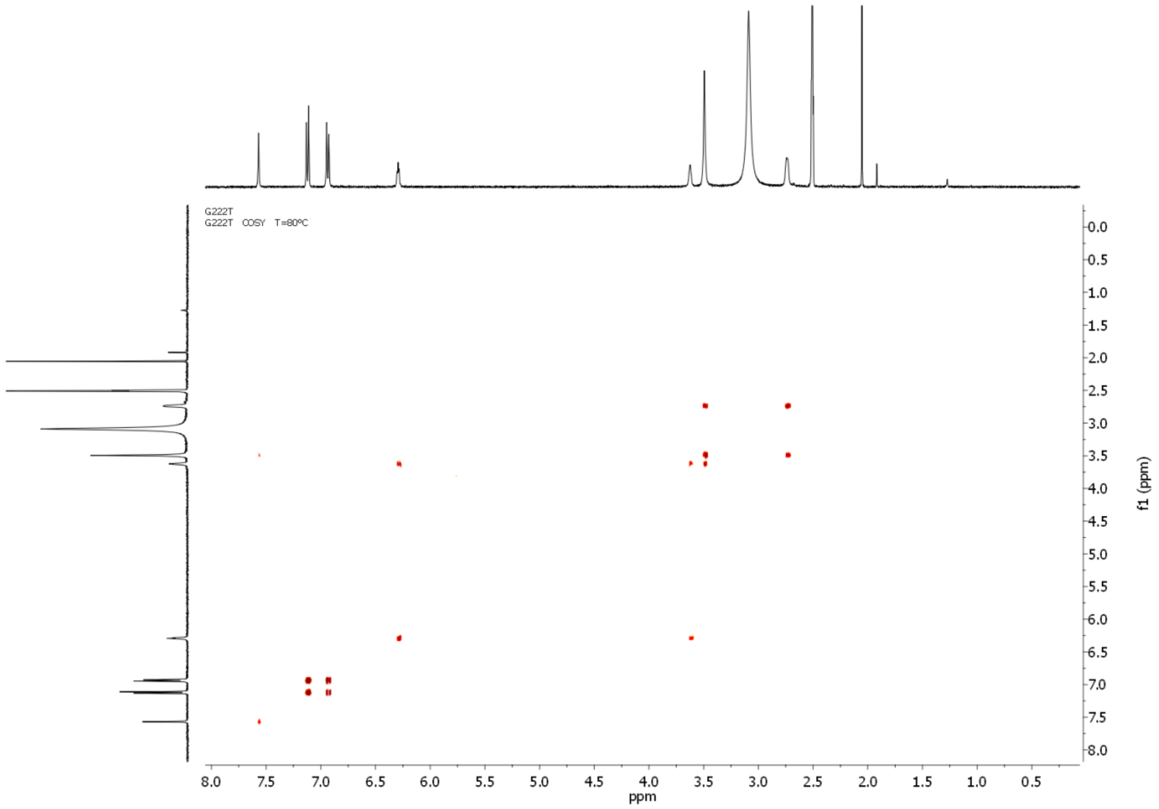

Fig. S32: COSY spectrum (d^6-DMSO) of **C1** at 80 °C

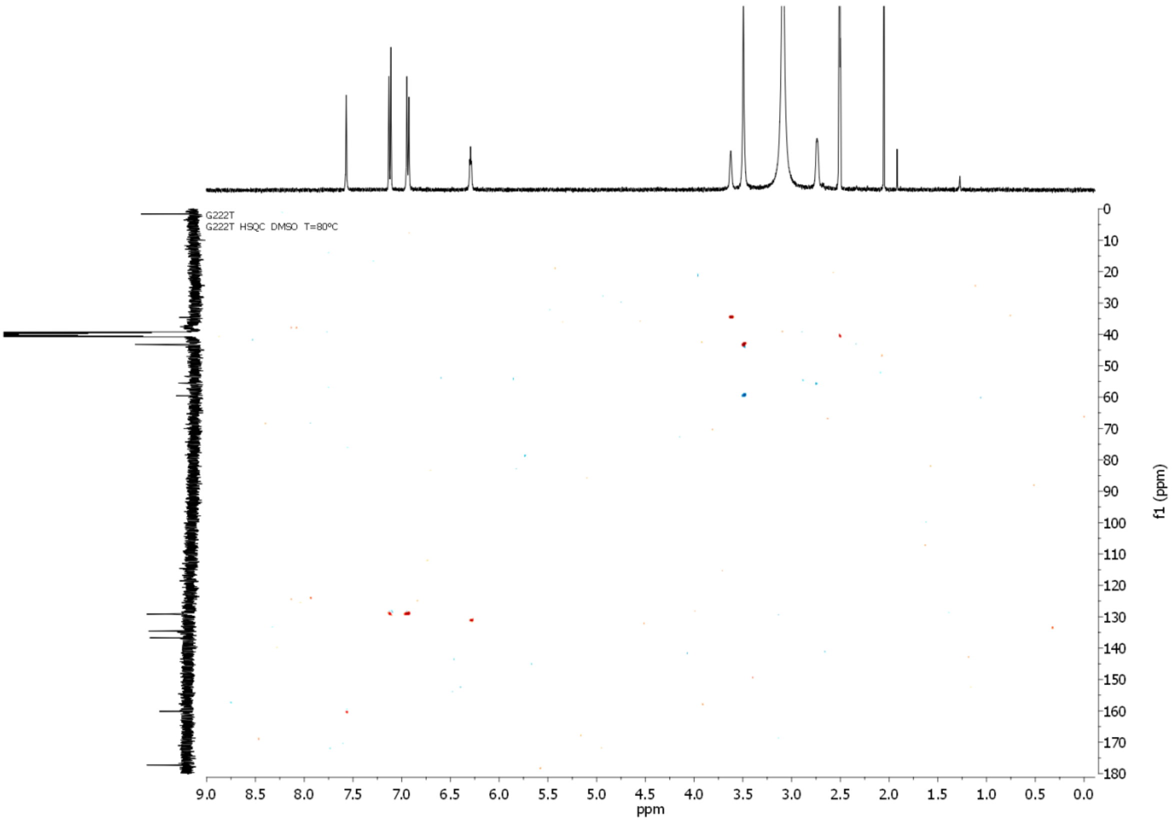

Fig. S33: HSQC spectrum (d^6-DMSO) of **C1** at 80 °C

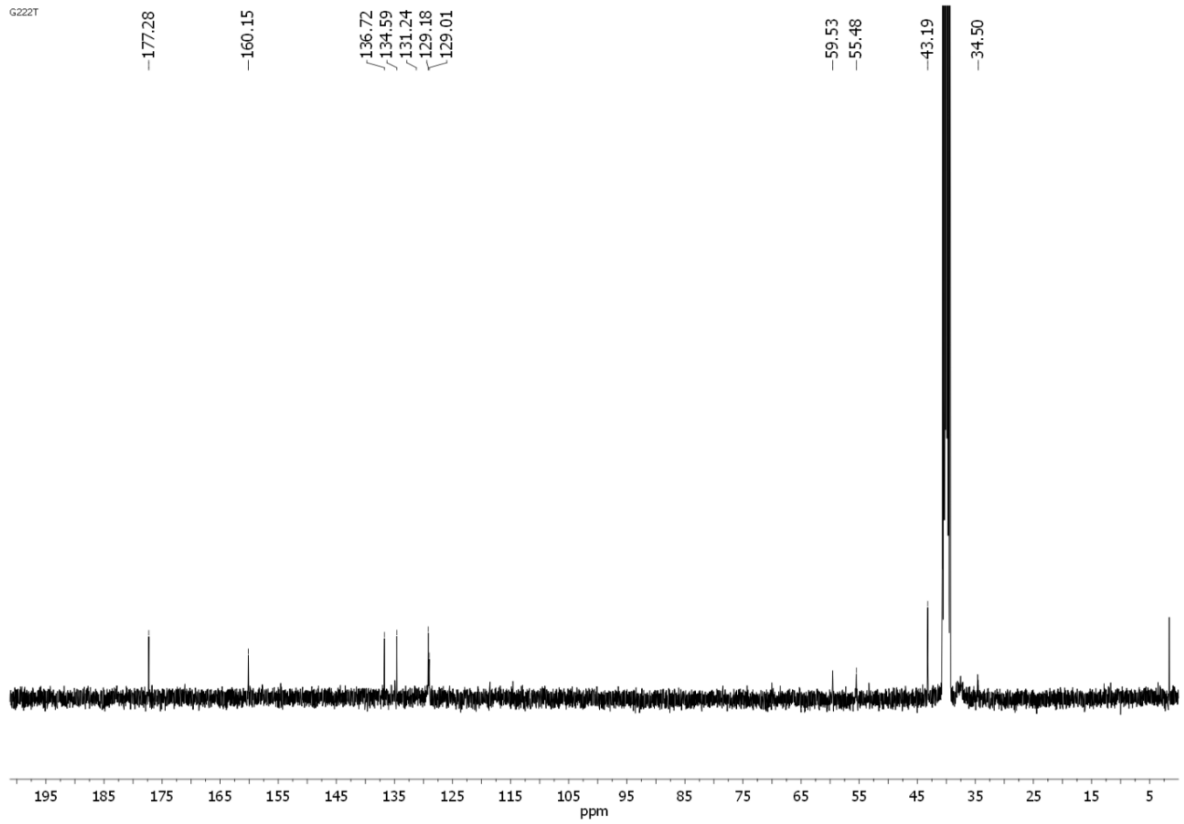

Fig. S34: ^{13}C-NMR spectrum (100 MHz; DMSO-d6) of **C1** at 80 °C

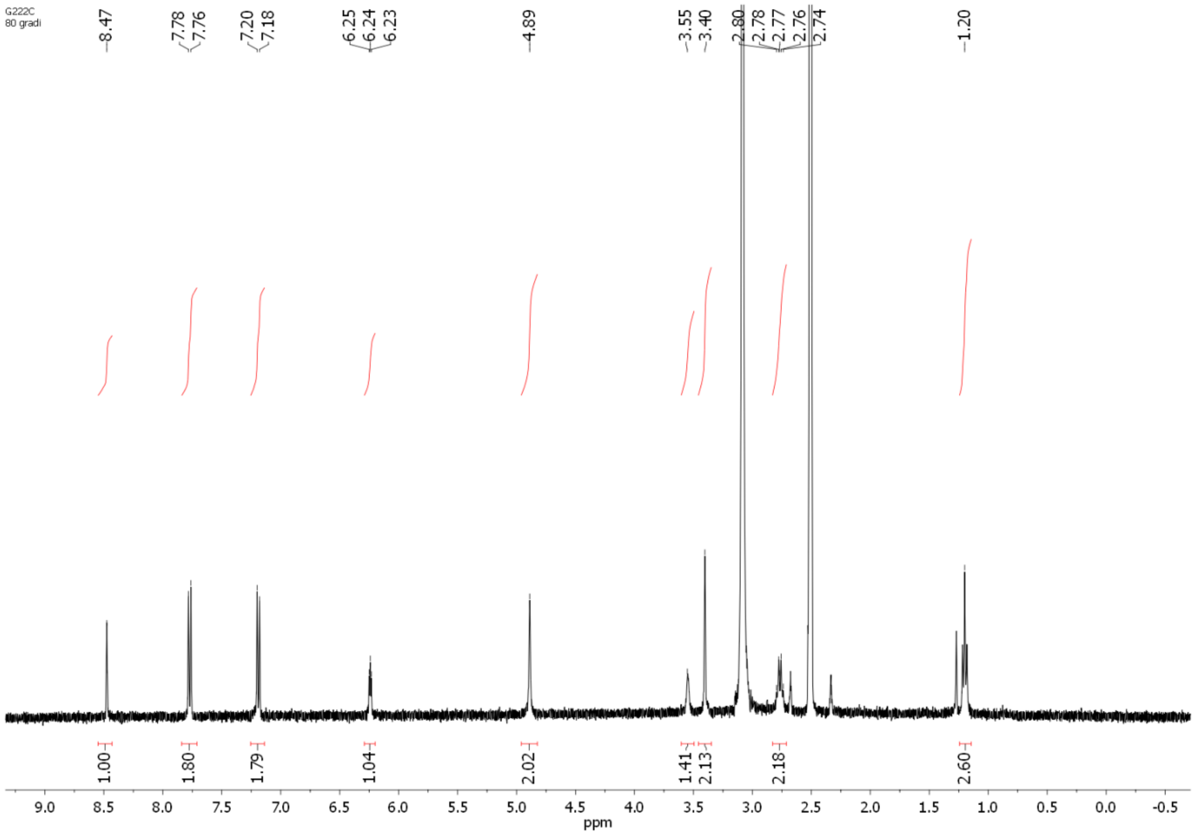

Fig. S35: ^{1}H-NMR(400 MHz; d^{6}-DMSO) of **C2** at 80 °C

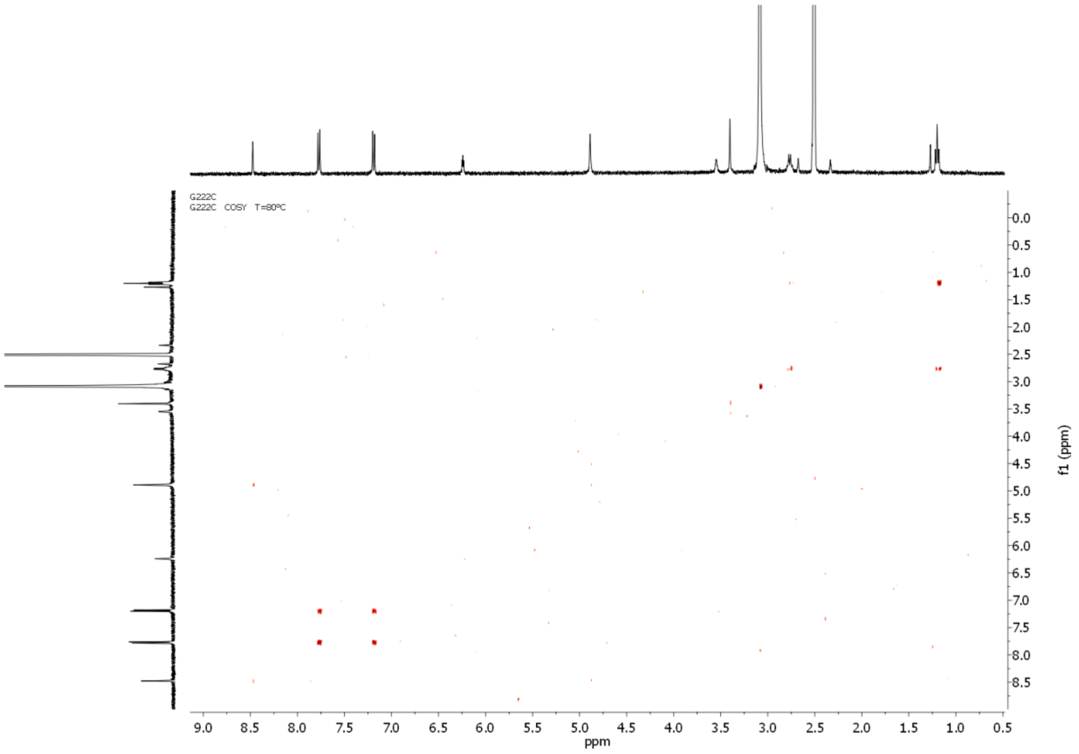

Fig. S36: COSY (400 MHz; d[6]-DMSO) of **C2** at 80 °C

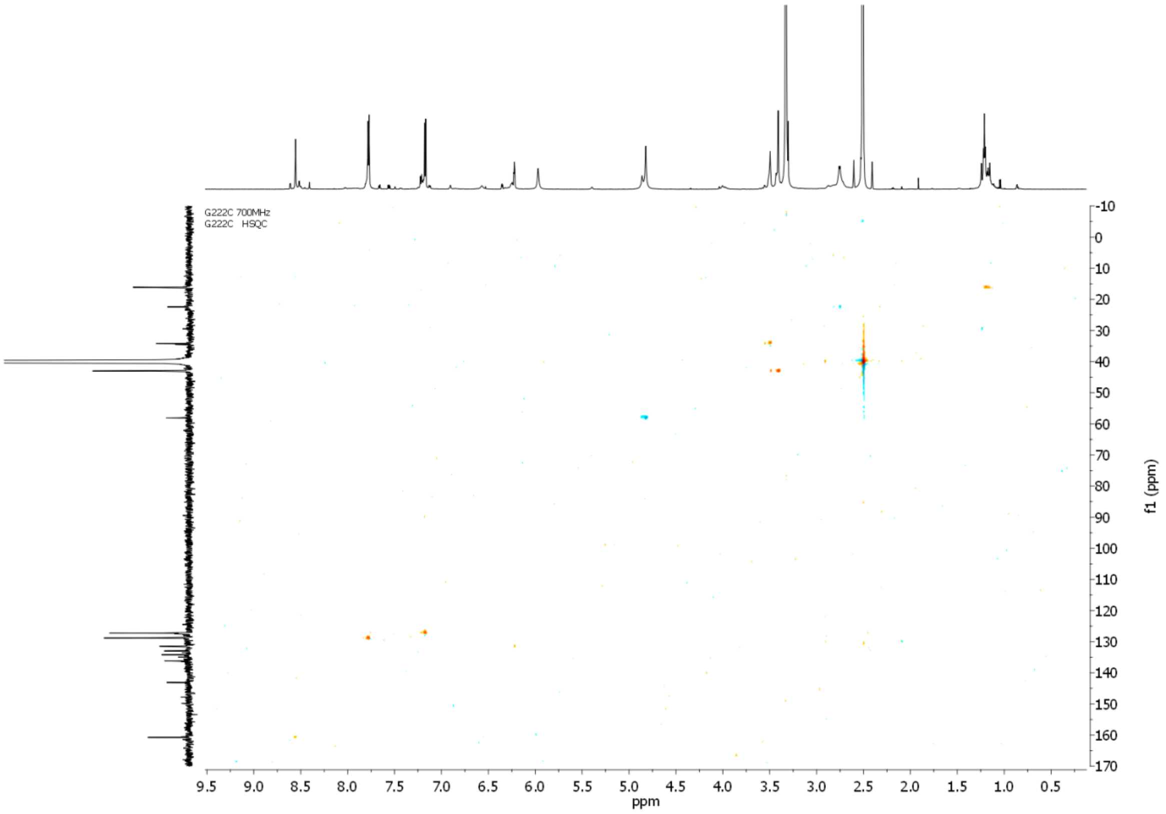

Fig. S37: HSQC spectrum(d[6]-DMSO) of **C2**

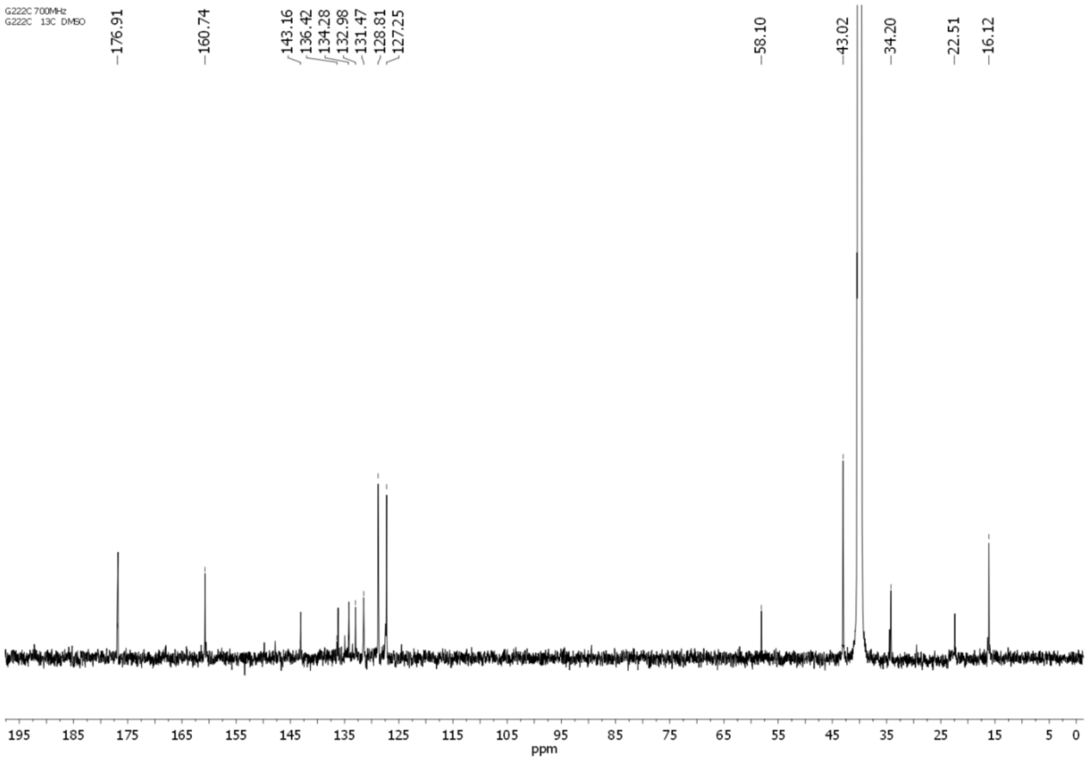

Fig. S38: ^{13}C-NMR (175 MHz; d^6-DMSO) of cage **C2**

Additional references

[1] S. I. Etkind, D. A. Vander Griend, T. M. Swager, *J. Org. Chem.* **2020**, **85**, 10050−1006. DOI:10.1021/acs.joc.0c01206.

[2] M. Monteleone, R. Mobili, C. Milanese, E. Esposito, A. Fuoco, S. La Cognata, V. Amendola, J.C. Jansen, *Molecules* **2021**, *26*, 5557. DOI:10.3390/molecules26185557.

[3] SAINT Software Reference Manual, Version 6, Bruker AXS Inc. **2003**, Madison, Wisconsin, USA.

[4] APEX 2, Bruker AXS Inc. **2012**, Madison, Wisconsin, USA.

[5] L. Krause, R. Herbst-Irmer, G. M. Sheldrick, D. Stalke, *J. Appl. Cryst.* **2015**, *48*, 3–10. DOI:10.1107/S1600576714022985.

[6] M.C. Burla, R. Caliandro, B. Carrozzini, G. L. Cascarano, C. Cuocci, C. Giacovazzo, M. Mallamo, A. Mazzone, G. Polidori, *J. Appl. Cryst.* **2015**, *48*, 306–309. DOI:10.1107/S1600576715001132.

[7] G. M. Sheldrick, *Acta Cryst.* **2015**, A*71*, 3–8. DOI:10.1107/S2053273314026370.

[8] G. M. Sheldrick, *Acta Cryst.* **2015**, C*71*, 3–8. DOI:10.1107/S2053229614024218.

[9] A. Lausi, M. Polentarutti, S. Onesti, J. R. Plaisier, E. Busetto, G. Bais, L. Barba, A. Cassetta, G. Campi, D. Lamba, et al., Eur. Phys. J. Plus 2015, 130, DOI 10.1140/epjp/i2015-15043-3.

[10] W. Kabsch, *Acta Cryst.* **2010**, D*66*, 125-132. DOI:10.1107/S0907444909047337.

[11] O. V. Dolomanov, L. J. Bourhis, R. J. Gildea, J. A. K. Howard, H. Puschmann, *J. Appl. Cryst*. **2009**, *42*, 339-341. DOI:10.1107/S0021889808042726.

[12] C. F. Macrae, I. J. Bruno, J. A. Chisholm, P. R. Edgington, P. McCabe, E. Pidcock, L. Rodriguez-Monge, R. Taylor, J. Van De Streek, P. A. Wood, J. Appl. Crystallogr. 2008, 41, 466–470.

[13] A. L. Myers, J. M. Prausnitz, *AlChE J.* **1965**, *11*, 121–127. DOI:10.1002/aic.690110125.

[14] M. Kang, S. Yoon, S. Ga, D. W. Kang, S. Han, J. H. Choe, H. Kim, D. W. Kim, Y. G. Chung, C. S. Hong, *Adv. Sci*. **2021**, *8*, 2004940, DOI: 10.1002/advs.202004940; X. Lv, L. Li, S. Tang, C. Wang, X. Zhao, *Chem. Commun*. **2014**, *50*, 6886–6889, DOI:10.1039/C4CC00334A.

[15] S. Lee, J. H. Lee, J. Kim, *Korean J. Chem. Eng*. **2018**, *35*, 214–221. DOI:10.1007/s11814-017-0269-9.

[16] H. Huang, K. Seob Song, A. Prescimone, A. Aster, G. Cohen, R. Mannancherry, E. Vauthey, A. Coskun, T. Šolomek, *Chem. Sci*. **2021**, *12,* 5275–5285. DOI:10.1039/d1sc00347j.

[17] F. Wang, E. Sikma, Z. Duan, T. Sarma, C. Lei, Z. Zhang, S. M. Humphrey, J. L. Sessler, *Chem. Commun*., **2019**, *55,* 6185–6188. DOI:10.1039/c9cc02490e.

[18] S. M. Elbert, F. Rominger, M. Mastalerz, *Chem. Eur. J*. **2014**, *20*, 16707–16720. DOI:10.1002/chem.201404829.

[19] D. Beaudoin, F. Rominger, M. Mastalerz, *Angew. Chem. Int. Ed*. **2017**, *56*, 1244–1248. DOI:10.1002/anie.201610782.

[20] X. Hu, W. Zhang, F. Rominger, I. Wacker, R. R. Schröder, M. Mastalerz, *Chem. Commun*. **2017**, *53*, 8616–8619. DOI:10.1039/c7cc03677a.

[21] B. Shimekit, H. Mukhtar, T. Murugesan, *J. Membr. Sci*. **2011**, *373*, 152–159. DOI:10.1016/j.memsci.2011.02.038.

[22] R. Pal, *J. Colloid Interface Sci.* **2008**, **317**, 191–198. DOI:10.1016/j.jcis.2007.09.032.

[23] S. C. Fraga, M. Monteleone, M. Lanč, E. Esposito, A. Fuoco, L. Giorno, K. Pilnáček, K. Friess, M. Carta, N. B. McKeown, P. Izák, Z. Petrusová, J. G. Crespo, C. Brazinha, J. C. Jansen, *J. Memb. Sci*. **2018**, *561,* 39–58. DOI:10.1016/j.memsci.2018.04.029.

[24] J. G. Wijmans, R. W. Baker in *Materials Science of Membranes for Gas and Vapor Separation* (Eds: Y. Yampolskii, Pinnau, I and Freeman, BD), John Wiley and Sons, Chichester, West Sussex, England **2006**, pp. 159–189.
